# Study protocol for first national vasa previa perinatal registry

**DOI:** 10.1371/journal.pone.0355045

**Published:** 2026-08-17

**Authors:** Kayla E. Paulosky, Antonio Santos-Roca, Chiara Corbetta-Rastelli, Efe Cudjoe, David Esterquest, Oluwaseun Fadairo, Brittany A. Fickau, Brenna Funfar, Allison Perelman, Naima Ross, Carrie A. Sibbald, Logan Todhunter, Bethany T. Waites, Andrew H. Chon, Chelsea A. DeBolt, Kathryn Drennen, Laura Hanks, Angie C. Jelin, Michelle Kush, Tara Lynch, Amol Malshe, Lauren A. Miller, Jessian L. Munoz, Mary E. Norton, Monica Rincon, Ashley S. Roman, Melissa Russo, Rachelle St. Onge, Yinka Oyelese, Guy Steinberg, Marika Toscano

**Affiliations:** 1 Department of Gynecology & Obstetrics, Johns Hopkins University School of Medicine, Baltimore, Maryland, United states of America; 2 Department of Obstetrics and Gynecology, University of Rochester Medical Center, Rochester, New York, United states of America; 3 Department of Obstetrics, Gynecology, and Reproductive Sciences, University of California San Francisco, San Francisco, California, United states of America; 4 Department of Obstetrics and Gynecology, Women & Infants Hospital of Rhode Island, Providence, Rhode Island, United states of America; 5 Department of Obstetrics and Gynecology, Advocate Lutheran General Hospital, Park Ridge, Illinois, United states of America; 6 Department of Obstetrics and Gynecology, Albany Medical Center, Albany, New York, United states of America; 7 Department of Obstetrics and Gynecology, Cleveland Clinic Akron General, Akron, Ohio, United states of America; 8 Department of Obstetrics and Gynecology, Jacobs School of Medicine and Biomedical Sciences, University at Buffalo, Buffalo, New York, United states of America; 9 Department of Obstetrics, Gynecology, and Reproductive Science, Icahn School of Medicine at Mount Sinai, New York, New York, United states of America; 10 Department of Obstetrics and Gynecology, NYU Grossman School of Medicine, New York, New York, United states of America; 11 Department of Obstetrics and Gynecology, University of Wisconsin School of Medicine and Public Health, Madison, Wisconsin, United states of America; 12 Department of Obstetrics and Gynecology, University of Colorado Anschutz School of Medicine, Aurora, Colorado, United states of America; 13 Department of Obstetrics and Gynecology, Oregon Health & Science University, Portland, Oregon, United states of America; 14 Department of Obstetrics and Gynecology, Baylor College of Medicine, Houston, Texas, United states of America; 15 Department of Obstetrics and Gynecology, Beth Israel Deaconess Medical Center, Boston, Massachusetts, United states of America; 16 Department of Obstetrics and Gynecology, Advocate Medical Group/Advocate Health, Downers Grove, Illinois, United states of America; Baylor College of Medicine, UNITED STATES OF AMERICA

## Abstract

Vasa previa is a rare but potentially catastrophic obstetric condition characterized by unprotected fetal blood vessels over or adjacent to the internal cervical os. Although advances in prenatal ultrasound have dramatically improved neonatal survival through planned cesarean delivery, many aspects of vasa previa remain poorly understood. Existing studies are limited by small sample sizes, single-center designs, heterogeneous diagnostic criteria, and inconsistent reporting of clinical outcomes, highlighting the need for large, systematically collected multicenter datasets. Here we describe, to our knowledge, the first United States multicenter registry of pregnancies complicated by vasa previa without concurrent placenta previa, the U.S. Vasa Previa Registry (US-VPR). This report outlines the rationale, design, and implementation of a large, multicenter, retrospective registry developed to characterize patient demographics, risk factors, placental pathology, natural history, antenatal management, maternal outcomes, neonatal outcomes, and healthcare utilization associated with vasa previa. The US-VPR includes contributions from 15 tertiary referral centers and represents the largest multicenter cohort of pregnancies complicated by vasa previa without concurrent placenta previa reported to date. By leveraging multicenter collaboration and standardized data collection, the registry provides a unique opportunity to characterize variation in clinical practice and outcomes across referral centers, strengthen the evidence base for this rare condition, and inform future prospective studies and evidence-based patient counseling.

## Introduction

Vasa previa is a rare but potentially catastrophic obstetric condition affecting ~1 in 1200 pregnancies [1]. It is defined by fetal blood vessels traversing the amniotic membranes over or adjacent to the internal cervical os, leaving them vulnerable to compression or rupture during labor or membrane rupture, which can result in rapid fetal exsanguination and acute fetal compromise [2,3]. Historically, undiagnosed vasa previa was associated with perinatal mortality rates exceeding 40%; however, advances in prenatal ultrasound diagnosis followed by planned prelabor cesarean delivery have improved neonatal survival to nearly 100% [4–12]. Approximately 80% of cases of vasa previa are associated with one or more identifiable risk factors, including velamentous cord insertion, second-trimester low-lying placenta, or placenta previa, conception via assisted reproductive technologies, bilobed or succenturiate placentas and multifetal gestation [11,13–17]. These factors facilitate prenatal detection during the second-trimester anatomy ultrasound, where transvaginal ultrasound is recommended for confirmation when vasa previa is suspected [5,18–23].

Vasa previa is commonly categorized into three anatomic subtypes based on underlying placental and cord anatomy; however, differences in clinical presentation, natural history and outcomes among these subtypes remain incompletely understood [24–26]. In addition, important controversies persist regarding the optimal definition and management of vasa previa. Debate continues regarding the distance from the internal cervical os required to establish the diagnosis, predictors of spontaneous resolution, the role of inpatient versus outpatient management, surveillance strategies, and the optimal timing of delivery [27–31]. Although recent internal expert consensus has provided guidance on several of these issues, many recommendations remain based on limited observational evidence rather than large, systematically collected datasets [3,32,33].

Similarly, the natural history of vasa previa across gestation remains poorly defined. Reported rates of spontaneous resolution range from approximately 6% to 39%, with higher rates observed when diagnosis occurs earlier in gestation [9,10,34–36]. These estimates are limited by small sample sizes, heterogeneous imaging protocols, and inconsistent definitions of resolution. Data identifying predictors of preterm delivery or persistent disease remain limited [35].

Although prenatal detection has markedly improved survival, antenatal management strategies for vasa previa vary widely across institutions [37,38]. Contemporary reviews and professional society guidance acknowledge limited evidence to inform decisions regarding surveillance intensity, inpatient versus outpatient management, and optimal timing of delivery [39,40]. Recent cohort studies demonstrate excellent survival among prenatally diagnosed cases but continue to reveal substantial heterogeneity in management approaches and resource utilization [41–43]. In parallel, an international Delphi survey of 68 experts reached consensus on several aspects of definition, screening, and clinical management; however, the evidence base informing these recommendations remains constrained by small cohorts and meta-analyses with inherent limitations [44].

In addition to management variability, neonatal outcomes associated with vasa previa have been incompletely described. Prior studies and meta-analyses have focused primarily on gestational age at delivery, birthweight, Apgar scores, neonatal anemia, transfusion requirements, and mortality [12,39,45]. Less is known about long-term postnatal clinical courses, neonatal intensive care unit utilization, and broader health system impacts. Concurrently, recent epidemiologic data suggest that vasa previa may be more common than previously recognized, underscoring the need for improved understanding of its clinical and resource implications in contemporary practice [1,19].

Despite substantial improvements in prenatal diagnosis and neonatal survival, the evidence base informing contemporary management remains constrained by the rarity of the condition. Most published studies consist of single-center experiences or relatively small retrospective cohorts with heterogeneous diagnostic criteria, inconsistent phenotyping, and variable management protocols, limiting comparisons across studies and reducing the generalizability of findings [33,46–49]. Consequently, important questions remain regarding risk factors, disease progression, recurrence risk, antenatal management, maternal morbidity, psychosocial impacts, neonatal outcomes, and healthcare resource utilization [46,50–55].

To address these persistent gaps, we established the United States Vasa Previa Registry (US-VPR), a multicenter registry of pregnancies complicated by vasa previa without concurrent placenta previa. The registry currently includes 15 institutions of varying sizes across the United States, providing one of the largest contemporary cohorts of patients with standardized clinical data collection. Prior to this effort, the largest single cohort reporting granular clinical details of vasa previa included 155 patients [18]. Our objective was to comprehensively characterize patient demographics, risk factors, placental pathology, natural history, recurrence risk, antenatal management strategies, maternal outcomes, neonatal outcomes, and healthcare utilization associated with vasa previa [39,56]. Our multicenter registry provides an opportunity to comprehensively characterize vasa previa across diverse practice settings using standardized data collection. These data will strengthen the evidence base for this rare but high-risk condition, inform future clinical management, and improve patient counseling, and ultimately optimize maternal and neonatal outcomes.

## Methods

### Rationale for a registry

Over the past decade, patient registries have become increasingly common across medicine because they are uniquely positioned to address important questions that cannot be adequately answered by randomized controlled trials (RCTs) or small observational studies alone.

Although RCTs remain the gold standard for evaluating the efficacy of specific interventions, they are poorly suited to the study of rare obstetric conditions such as vasa previa. The low incidence of vasa previa makes prospective randomized studies impractical, and existing cohorts are typically limited by small sample sizes, single-center designs, and heterogeneous inclusion criteria. Moreover, RCTs are not designed to characterize the natural history of disease, evaluate variation in real-world clinical management, assess patient experience, or quantify healthcare utilization and system-level burden. As a result, many critical aspects of vasa previa—including risk stratification, antenatal management strategies, timing of delivery, maternal outcomes, neonatal morbidity, and resource use—remain incompletely understood.

Patient registries are designed to capture large volumes of data generated during routine clinical practice without altering clinical care. By enrolling patients across diverse institutions and practice settings, registries enable comprehensive characterization of disease epidemiology, clinical heterogeneity, and real-world outcomes. For rare conditions, registries provide sufficient sample size to study uncommon presentations, management strategies, and adverse events that no single institution encounters frequently enough to evaluate independently. In addition, registries allow for the assessment of variation in clinical practice, generation of hypotheses for future prospective studies, and evaluation of healthcare utilization and costs—areas that are largely inaccessible through traditional clinical trials.

For vasa previa specifically, a national registry offers an opportunity to address persistent gaps in the literature. Baseline registry data can provide detailed information on patient demographics, obstetric history, placental and cord abnormalities, imaging findings, and risk factors associated with vasa previa. Longitudinal follow-up can elucidate the natural history of the condition, rates of persistence or apparent resolution, recurrence risk, and real-world antenatal management strategies, including inpatient versus outpatient care and timing of delivery. Importantly, a registry can also capture maternal outcomes, neonatal outcomes beyond survival alone, and healthcare utilization such as hospital admissions, length of stay, and neonatal intensive care unit use—outcomes that remain underreported in existing studies.

By leveraging multicenter collaboration and standardized data collection across institutions, the US-VPR is designed to provide comprehensive, real-world evidence on pregnancies complicated by vasa previa. Findings from this registry will strengthen the evidence base for vasa previa, inform future prospective studies, enhance patient counseling, and support the development of evidence-based approaches to the management of this rare but high-risk obstetric condition.

## Initial implementation

### Registry design and setting

This study was designed as a multicenter, retrospective, observational perinatal registry–style cohort of pregnancies complicated by vasa previa without concurrent placenta previa from January 1, 2011, through May 1, 2023. To date, the registry includes 15 U.S. medical centers (Table 1), each with established maternal–fetal medicine divisions and advanced obstetric imaging capabilities. Participating sites include tertiary and quaternary referral centers that care for both routine and high-risk obstetric populations. Although the majority of sites are academic institutions, several serve broad regional catchment areas and function as safety-net providers for medically complex pregnancies.

**Table 1:** Participating Institutions in the Vasa Previa Multicenter Registry.

Name	Location	Number of patients
Advocate Aurora Health (now Advocate Health)	Park Ridge, Illinois	41
Albany Medical Center	Albany, NY	14
Baylor College of Medicine	Houston, TX	Ongoing data collection
Beth Isreal Lahey Health	Boston, MA	Ongoing data collection
Warren Alpert Medical School of Brown University Women & Infants Hospital of Rhode Island	Providence, RI	Ongoing data collection
University at Buffalo (State University of New York)	Buffalo, NY	24
Cleveland Clinic	Cleveland, OH	81
University of Colorado Anschutz Medical Campus	Aurora, CO	68
Oregon Health & Science University	Portland, OR	28
University of Rochester Medical Center	Rochester, NY	65
Icahn School of Medicine at Mount Sinai	New York, NY	68
University of California, San Francisco	San Francisco, CA	112
New York University Grossman School of Medicine	New York, NY	97
University of Wisconsin School of Medicine and Public Health	Madison, WI	47
Johns Hopkins University School of Medicine	Baltimore, MD	95

This table is comprised of all current participating institutions in the Vasa Previa Multicenter Registry and their respective locations. This also highlights the number of patients obtained on initial chart review from each medical institution to date.

At the time of manuscript submission, the registry includes 15 participating institutions. Data collection has been completed at 12 sites, with continued accrual ongoing at 3 sites and anticipated to be completed by July 31, 2026. Data analysis has not yet begun and is expected to commence on May 1, 2026, with initial results anticipated by September 1, 2026.

**Fig 1:**
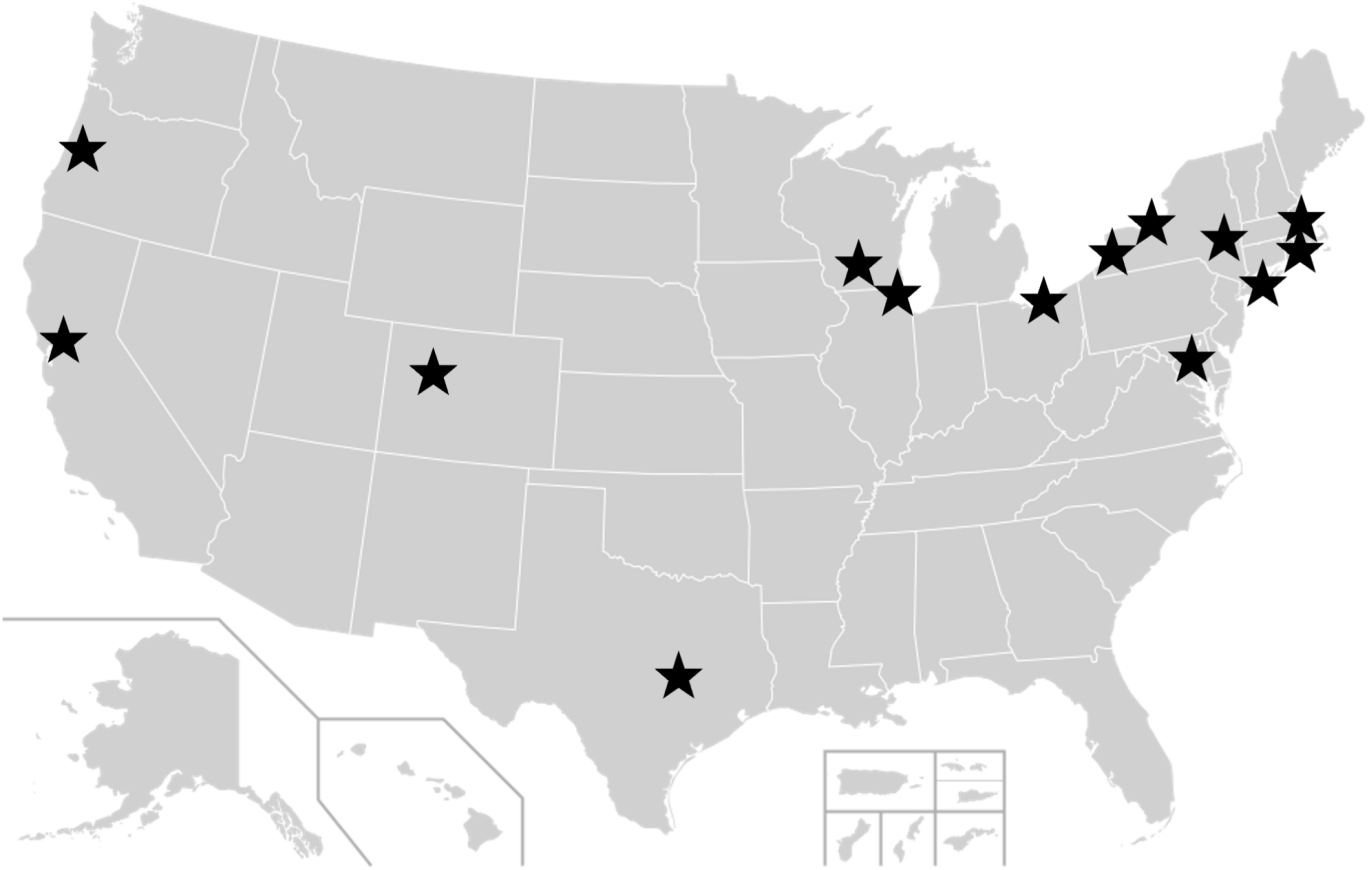
Geographic Distribution of Participating Institutions in the Vasa Previa Multicenter Registry. The stars represent the participating institutions. This basemap is public domain from Wikimedia Commons and is made available under the Creative Commons CC0 1.0 Universal Public Domain Dedication which is compatible with CC BY 4.0. https://commons.wikimedia.org/wiki/File:Blank_US_Map_(states_only).svg.

Participating institutions are geographically distributed across the Northeast, Midwest, West Coast, and Southern United States, supporting inclusion of a diverse patient population and a range of practice patterns (Fig 1).

Recruitment of centers occurred through professional networks in obstetrics and maternal–fetal medicine, direct outreach to institutions with expertise in high-risk pregnancy care, and through review of presentations at national and regional scientific meetings. Informal professional referral and word-of-mouth also contributed to recruitment. Institutional participation requirements are displayed in Table 2**.**

**Table 2:** Institution participation requirements.

Institution Participation Requirements
1. Elected to participate in registry voluntarily 2. Obtained institutional review board approval at respective institution 3. Completed required data use and collaboration agreement 4. Completed case ascertainment at institution through methods determined by local study personnel 5. Completed and validated electronic medical record data extraction

All institutions had to adhere to the following requirements prior to joining the national registry of vasa previa patients. There was no minimum patient contribution requirement to participate.

This initial formation of the US-VPR registry is retrospective in nature, capturing historical cases identified through existing obstetric ultrasound databases, electronic health records, and billing systems. No interventions were introduced as part of the study; all care was delivered per local clinical practice, rendering the registry purely observational.

The registry is health-system–based, drawing from large academic and tertiary/quaternary referral centers with specialized maternal–fetal medicine ultrasound services rather than from population-level surveillance systems. This design was selected to enable detailed phenotyping, imaging review, placental pathology assessment, and granular neonatal outcome ascertainment for a rare obstetric diagnosis.

Johns Hopkins University serves as the central coordinating and data management center for the registry. Oversight was provided by the study principal investigator in collaboration with designated site principal investigators at each participating institution. A central governance structure was implemented to promote data consistency and scientific rigor. This included: (1) Standardized inclusion and exclusion criteria; (2) Uniform variable definitions and data dictionaries; (3) Centralized data storage in a secure REDCap database housed at the coordinating center.

Each participating institution retained responsibility for local regulatory oversight, including Institutional Review Board (IRB) approval and protection of protected health information, and data use agreements were executed between institutions prior to data sharing. During the process of case identification and chart abstraction at participating institutions, site investigators had access to identifiable patient information within their local electronic health records. Only de-identified data were subsequently transferred to the coordinating center for analysis, and the study team at the coordinating site did not have access to direct patient identifiers. Data access was restricted to IRB-approved study personnel. The principal investigator convened regular investigator meetings to coordinate analysis and dissemination of findings. Proposed secondary analyses of the pooled, multicenter dataset arediscussed at monthly collaborative group meetings and compiled into a written document to facilitate group review, coordination, and documentation. All analyses are planned to be conducted by the same statistical team at the coordinating center to promote continuity and consistency across analyses.

Policies governing data use, analysis, and publication emphasized collaborative authorship, transparency, and scientific integrity.

### Study population

The US-VPR aims to capture data on pregnancies complicated by vasa previa across participating centers. As with all observational registries, some degree of unavoidable selection exists. First, inclusion is limited to pregnancies managed at institutions that have elected to participate in the registry, obtained local institutional review board (IRB) approval, and executed the required data use for collaboration agreement. Second, cases must be identified and diagnosed as vasa previa by the treating clinical team based on predefined diagnostic criteria. Third, data inclusion depends on successful case ascertainment and abstraction by local study personnel, as data entry is voluntary rather than mandatory at participating sites.

Beyond these practical considerations, the registry includes pregnancies with prenatal ultrasound diagnosis of vasa previa (fetal vessels ≤2 cm from the internal cervical os) or low-lying fetal vessels (>2–5 cm from the internal cervical os), delivery at a viable gestational age, and availability of complete maternal delivery and corresponding neonatal records. Pregnancies are eligible regardless of maternal age, race or ethnicity, insurance status, comorbidities, plurality, or treatment setting. Both singleton and multiple gestation pregnancies are eligible for inclusion. Pregnancies complicated by concurrent placenta previa at delivery are excluded, consistent with the registry’s design and analytic focus. However, patients that had placenta previa that resolved were included. Individuals with resolved vasa previa were included but will be analyzed as a separate cohort. Inclusion and exclusion criteria are summarized in Table 3.

**Table 3:** Eligibility for the vasa previa registry.

Inclusion Criteria	Exclusion Criteria
1. Pregnancies managed at participating institutions with IRB approval and data use agreements 2. Prenatal ultrasound diagnosis of vasa previa (fetal vessels ≤2 cm from the internal cervical os) or low-lying fetal vessels (>2–5 cm from the internal cervical os) 3. Delivery at viable gestational age including both live born or stillborn infants 4. Complete maternal delivery and neonatal records available 5. Singleton or multifetal gestation pregnancies 6. All maternal demographics and clinical characteristics eligible (no restrictions based on age, race/ethnicity, insurance status, or comorbidities) 7. Resolved vasa previa included but analyzed as a separate cohort	1. Concurrent placenta previa 2. Pregnancies without ultrasound records, delivery records or neonatal records

This table highlights the subject inclusion and exclusion criteria for the national vasa previa registry. All institutions were required to screen patients separately based off this criteria

### Population representativeness

This registry represents a health-system–based cohort drawn from large academic and tertiary/quaternary referral centers with specialized maternal–fetal medicine ultrasound services. As such, the study population reflects patients receiving care for both routine and high-risk pregnancies, including referrals for suspected or confirmed vasa previa. Because vasa previa is a rare obstetric condition that is frequently referred to tertiary/quaternary maternal–fetal medicine centers for diagnosis and management, this design enabled accrual of one of the largest contemporary cohorts while maximizing detailed clinical phenotyping and outcome ascertainment.

Although the cohort is not population-based, the multicenter design and broad geographic distribution of participating institutions enhance heterogeneity in patient demographics and clinical practice patterns. This design prioritizes depth of clinical phenotyping and outcome ascertainment for a rare obstetric condition over population-level incidence estimation.

Formal comparison to underlying source populations at each site was not performed; however, inclusion of all eligible cases identified during the study period at participating institutions minimizes selection bias within health systems.

## Data sources

### Primary data sources

The primary data source for this registry is the electronic health record (EHR) at each participating institution. Clinical data are derived from maternal and neonatal EHRs, including prenatal, delivery, and postpartum encounters.

EHR data are supplemented by institutional obstetric ultrasound databases, which served as the primary source for case identification and detailed characterization of vasa previa, cord insertion, placental morphology, and vessel location. These systems included structured ultrasound reporting platforms routinely used in maternal–fetal medicine practice.

To ensure comprehensive and accurate variable capture, data are retrospectively abstracted from the medical record by clinically-trained site personnel using standardized REDCap electronic data capture forms hosted at each participating institution. Identical REDCap data collection instruments using standardized variable definitions and data dictionaries developed by the coordinating center are used across sites to promote consistency in data abstraction. All data collection instruments are administered in English; however, data abstraction is performed by trained site personnel using the medical record, and no direct patient-facing questionnaires were required for inclusion in the present cohort. Upon completion of data collection, each institution exports its local REDCap data and securely transfers the dataset to the coordinating center, where data from all participating sites are imported into a single centralized REDCap database for data management and analysis.

### Data capture frequency and timepoints

Data collection for this initial registry creation has thus far been retrospective and episodic, rather than real-time. Each participating site performed a one-time identification and abstraction of all eligible cases occurring during the study period.

Clinical data are captured across key obstetric and neonatal timepoints, including:

- Antepartum: prenatal diagnosis, imaging findings, antenatal surveillance, inpatient versus outpatient management, and complications
- Delivery: gestational age, mode and timing of delivery, delivery indications, and operative findings
- Postpartum and neonatal period: placental pathology, immediate neonatal outcomes, neonatal intensive care unit (NICU) admission and course, and newborn discharge-related variables

No prospective follow-up or repeated data extraction was performed beyond what is available in the existing medical record for the index pregnancy and neonatal hospitalization.

### Participant identification

Pregnancies complicated by vasa previa included in the US-VPR were identified using a multi-step, confirmatory case ascertainment approach at each participating institution is shown in S2 Table. Initial case identification was performed through:

- Queries of institutional obstetric ultrasound databases used in routine maternal–fetal medicine practice, which contain structured documentation of placental location, cord insertion, and fetal vessel anatomy.
- Queries of hospital administrative and billing systems for ICD diagnosis codes associated with vasa previa.

These initial electronic queries were intentionally inclusive and were followed by manual chart review to confirm eligibility. A pregnancy was classified as affected by vasa previa or low-lying fetal vessels only if the diagnosis was documented on prenatal ultrasound imaging and confirmed by trained clinician-performed chart review. Administrative diagnosis codes (e.g., ICD-10) were used solely to aid case finding and were not relied upon in isolation to define exposure status. Final case adjudication was based on ultrasound findings and clinical documentation rather than billing codes alone.

### Dating of pregnancy

Gestational dating was derived from the best obstetric estimate documented in the medical record, consistent with standard clinical practice at participating institutions. Pregnancy start date was determined using the estimated gestational age assigned by the treating clinicians. Pregnancy end date was defined as the date of delivery. Pregnancies ending in fetal demise were dated based on the documented delivery or diagnosis date. Gestational age at key timepoints—including diagnosis, initiation of inpatient management, and delivery—was calculated using the recorded gestational age corresponding to those clinical encounters.

### Handling of complex scenarios

Several potential complexities inherent to registry-based pregnancy identification are addressed as follows:

- Early pregnancy loss: Pregnancies that did not progress to delivery at a viable gestational age are excluded by design, as eligibility required delivery at a viable gestational age with complete delivery records available. Viability was defined according to each local institutional neonatal resuscitation practices in effect at the time of care, recognizing that the threshold for viability is variable based on institution and time period.
- Stillbirth: Pregnancies complicated by stillbirth at or beyond viability were eligible for inclusion for ultrasound and antenatal management variables. Delivery and neonatal outcome variables may be collected, but will be excluded from analyses regarding delivery timing, mode of delivery, and neonatal outcomes.
- Transfers between facilities: For patients transferred between institutions, pregnancy episodes are attributed to the site responsible for delivery, with antenatal data abstracted from available records within the delivering institution’s EHR.
- Overlapping or conflicting records: In cases of discrepant documentation (e.g., differing descriptions of vessel location or placental morphology), ultrasound reports and specialist maternal–fetal medicine documentation are prioritized over administrative data. Ambiguous cases are resolved through chart review and individual image review by site investigators.

Each eligible pregnancy is treated as a distinct analytic episode, even if individuals contributed more than one pregnancy during the study period. This approach reflects the pregnancy-specific nature of vasa previa risk, diagnosis, and management.

### Variables of interest

The registry captures data across five core domains: maternal demographics and medical/obstetric history, ultrasound features of vasa previa, pregnancy complications, delivery characteristics, and neonatal outcomes, which were informed by prior publications [56,57]. Maternal demographic variables include age, race, ethnicity, and body mass index. Medical and obstetric history includes gravidity, parity, prior uterine surgery, and use of assisted reproductive technologies. Ultrasound variables include vasa previa subtype, distance from internal os, number of gestations, and placental and umbilical cord characteristics. Pregnancy complications and antenatal course includes resolution of vasa previa, antenatal management strategies, and antepartum bleeding events. Delivery characteristics include gestational age at delivery, mode and timing of delivery, and delivery indications. Neonatal outcomes include birthweight, Apgar scores, need for resuscitation or transfusion, NICU admission and length of stay, and major neonatal morbidities.

Outcome measures are defined using standardized, clinically accepted operational definitions where available and shown in S1 Table. Severe maternal morbidity is classified using Centers for Disease Control and Prevention (CDC) indicators. Neonatal outcomes are abstracted directly from the medical record using established clinical definitions. When applicable, outcome definitions were informed by prior studies and consensus guidelines.

Covariates include clinical factors (e.g., gestational age at diagnosis, plurality, cord insertion type), social determinants of health available in the medical record (e.g., race and ethnicity), and health system variables such as inpatient versus outpatient management and site of care. These variables were selected a priori based on biologic plausibility and relevance to antenatal management and neonatal outcomes in pregnancies affected by vasa previa.

A complete variable list, including variable definitions, is provided in S1 Table.

### Data quality assurance

Data collection is standardized across participating sites using a centralized data dictionary and prespecified variable definitions developed by the coordinating center. All data are entered into a registry-specific REDCap database, which served as the common data structure for the study. Prior to data entry, site investigators received guidance on variable definitions, inclusion criteria, and abstraction procedures to promote cross-site consistency.

Cross-site harmonization is supported through ongoing communication between the coordinating center and participating institutions, including regular investigator meetings to review data collection practices, clarify definitions, and address discrepancies identified during abstraction.

Data quality is assessed using a combination of automated and manual checks. Built-in REDCap functionality is used to implement range checks (e.g., gestational age, birthweight) and logic checks (e.g., delivery date occurring after diagnosis date; gestational age consistency across timepoints).

Patterns of missingness are monitored at the site level, and sites are contacted to resolve missing or implausible values when identified. No formal missingness thresholds are used to exclude sites or cases; instead, missing data will be addressed descriptively in analysis.

All cases included in the registry undergo manual chart review to confirm eligibility and abstract key variables. For pregnancy identification, concordance between administrative data, ultrasound reports, and clinical documentation was assessed during chart review, with prenatal ultrasound findings prioritized for confirmation of vasa previa diagnosis and classification.

Administrative diagnosis codes are used solely for case-finding and are not relied upon to define exposure status. As such, formal algorithm validation metrics are not calculated. Ultrasound-based diagnostic criteria and outcome definitions were informed by prior literature and established clinical practice guidelines.

### Ethical considerations

This study was reviewed and approved by the Institutional Review Board (IRB) at the original site, which served as the coordinating IRB for the multicenter study. Participating institutions obtained local IRB approval in accordance with their institutional policies.

Given the retrospective design and exclusive use of existing clinical data, the IRB granted the registry a waiver of informed consent and a waiver of HIPAA authorization. The waivers were granted on the basis that the research posed no more than minimal risk to participants, could not practicably be conducted without access to protected health information, and involved no direct contact with participants. Data was not anonymized when it was initially accessed but the data was deidentified during the data collection process and prior to data transfer to the coordinating center.

### Statistical analysis and timeline

This report is primarily descriptive and focuses on the design of the registry and baseline characteristics of the included institutions. The initial set of investigators of the US-VPR have identified several planned analyses using the data collected. The initial publication will focus on describing the baseline characteristics of the registry cohort, including patient demographics, obstetric history, placental and cord abnormalities, imaging findings, and antenatal management practices. Subsequent analyses will examine clinically relevant subgroups, including comparisons by vasa previa subtype (Types I, II, and III), distance of fetal vessels from the internal cervical os, and inpatient versus outpatient management strategies.

Planned analyses will also evaluate differences in maternal outcomes, neonatal outcomes, and healthcare utilization across these subgroups, with the goal of identifying patterns that may generate hypotheses regarding risk stratification and individualized management. Together, these studies aim to clarify heterogeneity in presentation, management, and outcomes among pregnancies complicated by vasa previa and to provide an evidence base to support more tailored counseling and care.

### Handling of missing data

Given the retrospective design, missing data are anticipated for certain variables. Analyses will be conducted using a complete-case approach for variables of interest, with the extent and distribution of missing data, including variation by site, reported descriptively. No formal imputation procedures are planned, as the primary intent of the registry is characterization rather than causal inference.

## Discussion

### Strengths and limitations of the registry

This registry represents one of the largest multicenter cohorts of pregnancies complicated by vasa previa without concurrent placenta previa, a rare obstetric condition that has historically been limited to small case series. The multicenter design, inclusion of geographically distributed tertiary/quaternary referral centers, and extended study period enhance the breadth of clinical practice patterns and outcome ascertainment.

A key strength of the registry is the use of prenatal ultrasound confirmation and electronic medical record review for case adjudication, rather than reliance on administrative codes alone, improving diagnostic accuracy. Additionally, all cases meeting criteria undergo a multilevel adjudication process, first by the individual institutions and then validated by the coordinating center. The registry includes granular antenatal management data, detailed placental pathology findings, and comprehensive neonatal outcomes, enabling characterization of both clinical care processes and short-term neonatal morbidity.

Standardized data collection procedures, centralized data management, and manual chart review across sites further support data quality. The pregnancy-level analytic framework allows evaluation of natural history, management strategies, and recurrence risk, which are particularly relevant for counseling and clinical decision-making in vasa previa.

Several limitations should be considered when interpreting findings from this registry. The retrospective design introduces the potential for missing data, misclassification, and documentation variability across sites. Although all participating institutions adhered to the same inclusion and exclusion criteria, cases are identified using contemporaneous local clinical diagnoses rather than centralized prospective imaging review. Because the study spans more than a decade, evolving ultrasound technology, increasing awareness of vasa previa, and changing diagnostic definitions—including ongoing debate regarding the distance threshold from the internal cervical os and classification of low-lying fetal vessels—may contribute to temporal and institutional heterogeneity in case ascertainment. While all cases undergo multilevel adjudication by the participating site and coordinating center, these procedures can not completely eliminate variation in diagnostic practices.

The registry is health-system–based and largely composed of academic referral centers, which is consistent with the specialized care typically required for vasa previa, but may limit representation of cases managed outside participating referral networks and generalizability to lower-resource or non-referral settings. Population-level incidence estimates could not be derived, and formal comparisons to underlying source populations were not performed.

Long-term maternal and child outcomes beyond the neonatal hospitalization are not collected in this study, and patient-reported outcomes are not captured. Finally, although recurrence risk is assessed, follow-up is limited to subsequent pregnancies documented within participating health systems.

### Data access

Participating sites retain the right to independently analyze, present, and publish their own internal data without the approval of the US-VPR research group, on the condition that they acknowledge the US-VPR project’s role in the research design, data collection and project management. Secondary statistical analyses of the entire US-VPR database may also be proposed by any participating site who has contributed a minimum of 20 cases to the registry. Investigators may propose secondary analyses of the pooled, multicenter dataset for consideration by the collaborative group. Proposed analyses are discussed at monthly investigator meetings and compiled into a written document to facilitate group review, coordination, and documentation; assess scientific merit and feasibility; ensure alignment with registry priorities; and avoid overlap with planned or ongoing analyses. All analyses are planned to be conducted by the same statistical team at the coordinating center to promote continuity and consistency across projects. Analyses and dissemination of findings will be conducted in accordance with applicable registry governance, data use agreements, and authorship policies.

### Upcoming developments

The US-VPR was designed as a foundational platform with the capacity to evolve over time. A key upcoming development is a planned transition from a static, retrospective registry to a living registry with ongoing data accrual. Additional institutions that were not included in the initial registry would have the opportunity to contribute data once they receive IRB approval. At this time all participating institutions are located within the United States and there are no immediate plans for this to become an international registry due to funding limitations. This evolution will allow the registry ongoing capture of contemporary practice patterns, longitudinal outcomes, and changes in management strategies as evidence and clinical guidance continue to evolve.

## Supporting information

S1 Table Data Elements Collected in the Vasa Previa Without Concurrent Placenta Previa Multicenter Registry. (DOCX)

S2 Table Multistep Case Ascertainment Strategy for Identification of Vasa Previa. (DOCX)
