# Supplementary material for: Study protocol for first national vasa previa perinatal registry
Source: PLoS One. 2026 Aug 17;21(8):e0355045. doi: 10.1371/journal.pone.0355045 (PMC13480644; doi:10.1371/journal.pone.0355045)

PLOS One

Study Protocol for First National Vasa Previa Perinatal Registry

--Manuscript Draft--

**Manuscript Number:**	PONE-D-26-11238
**Article Type:**	Study Protocol
**Full Title:**	Study Protocol for First National Vasa Previa Perinatal Registry
**Short Title:**	Vasa Previa Perinatal Registry Protocol
**Corresponding Author:**	Marika Toscano<br>Johns Hopkins University<br>Baltimore, MD UNITED STATES OF AMERICA
**Keywords:**	Vasa previa<br><br>Low-lying fetal vessels<br><br>Perinatal registry<br><br>Multicenter registry<br><br>Observational cohort<br><br>Rare obstetric condition<br><br>Diagnosis/imaging<br><br>Prenatal diagnosis<br><br>Obstetric ultrasound<br><br>Transvaginal ultrasound<br><br>Velamentous cord inser
**Abstract:**	Vasa previa is a rare but potentially catastrophic obstetric condition characterized by unprotected fetal blood vessels over or adjacent to the internal cervical os. Although advances in prenatal ultrasound have dramatically improved neonatal survival through planned cesarean delivery, many aspects of vasa previa remain poorly understood. Existing studies are limited by small sample sizes, single-center designs, heterogeneous definitions, and variable reporting of maternal, neonatal, and health system outcomes. As a result, uncertainty persists regarding the natural history of vasa previa, optimal antenatal management strategies, patient experience, and resource utilization. Here we describe, to our knowledge, the first United States multicenter registry of pregnancies complicated by vasa previa without concurrent placenta previa, the U.S. Vasa Previa Registry (US-VPR). established to improve the scientific understanding, clinical management, and health system planning for pregnancies complicated by vasa previa. This is an ongoing, multicenter, observational registry consisting of institutions across the U.S. This report describes the rationale, design, and initial implementation of a large, multicenter registry focused on characterizing the demographics, risk factors, placental pathology, natural history, antenatal management strategies, maternal outcomes, neonatal outcomes, and healthcare utilization associated with vasa previa. Currently, the US-VPR includes contributions from 15 institutions representing the largest multicenter cohort of pregnancies complicated by vasa previa reported to date. The registry captures a substantially larger and more diverse population than previously reported single-center cohorts. It is designed to support ongoing analyses of clinical, patient-centered, and health system outcomes, as well as variation in management practices for vasa previa. By leveraging multicenter collaboration and standardized data collection, the registry provides a platform to generate high-quality, real-world evidence to inform clinical decision-making, guide individualized management strategies, improve patient counseling, and optimize maternal and neonatal outcomes. Continued participation and expansion of the registry

Powered by Editorial Manager® and ProduXion Manager® from Aries Systems Corporation

	will further enhance its ability to address unanswered questions and improve care for patients affected by vasa previa.
**Order of Authors:**	Kayla E. Paulosky, MD
	Chiara Corbetta-Rastelli, MD
	Efe Cudjoe, MD
	David Esterquest, MD
	Oluwaseun Fadairo, MD
	Brittany Fickau, MD
	Brenna Funfar, MD
	Allison Perelman, MD
	Naima Ross, MD
	Antonio Santos-Roca, MD
	Carrie Sibbald, MD
	Logan Todhunter, MD
	Bethany Waites, MD
	Andrew Chon, MD
	Chelsea A. DeBolt, MD
	Kathryn Drennen, MD
	Laura Hanks, MD
	Angie Jelin, MD
	Michelle Kush, MD
	Tara Lynch, MD
	Amol Malshe, MD
	Lauren Miller, MD
	Jessian Munoz, MD
	Mary Norton, MD
	Monica Rincon, MD
	Ashley S. Roman, MD
	Melissa Russo, MD
	Guy Steinberg, MD
	Rachelle St. Onge, MD
	Yinka Oyelese, MD
	Marika Toscano
**Additional Information:**	
**Question**	**Response**
**Financial Disclosure**<br><br>Enter a financial disclosure statement that describes the sources of funding for the work included in this submission. Review the submission guidelines for detailed	Yes

requirements. View published research articles from *PLOS ONE* for specific examples.<br><br>This statement is required for submission and **will appear in the published article** if the submission is accepted. Please make sure it is accurate.<br><br>**Funded studies**<br>Enter a statement with the following details:<br>• Initials of the authors who received each award<br>• Grant numbers awarded to each author<br>• The full name of each funder<br>• URL of each funder website<br>• Did the sponsors or funders play any role in the study design, data collection and analysis, decision to publish, or preparation of the manuscript?<br><br>Did you receive funding for this work?	
Please add funding details.<br>as follow-up to "**Financial Disclosure**<br><br>Enter a financial disclosure statement that describes the sources of funding for the work included in this submission. Review the submission guidelines for detailed requirements. View published research articles from *PLOS ONE* for specific examples.<br><br>This statement is required for submission and **will appear in the published article** if the submission is accepted. Please make sure it is accurate.	National Institutes of Health - 2K12AR084229-12<br><br>National Institutes of Health - 5K12HD103036

Powered by Editorial Manager® and ProduXion Manager® from Aries Systems Corporation

**Funded studies**<br>Enter a statement with the following details:<br>• Initials of the authors who received each award<br>• Grant numbers awarded to each author<br>• The full name of each funder<br>• URL of each funder website<br>• Did the sponsors or funders play any role in the study design, data collection and analysis, decision to publish, or preparation of the manuscript?<br><br>Did you receive funding for this work?"	
Please select the country of your main research funder (please select carefully as in some cases this is used in fee calculation).<br>as follow-up to "**Financial Disclosure**<br><br>Enter a financial disclosure statement that describes the sources of funding for the work included in this submission. Review the submission guidelines for detailed requirements. View published research articles from *PLOS ONE* for specific examples.<br><br>This statement is required for submission and **will appear in the published article** if the submission is accepted. Please make sure it is accurate.<br><br>**Funded studies**<br>Enter a statement with the following details:<br>• Initials of the authors who received each award<br>• Grant numbers awarded to each author<br>• The full name of each funder<br>• URL of each funder website<br>• Did the sponsors or funders play any role in the study design, data collection and analysis, decision to publish, or preparation of the manuscript?<br><br>Did you receive funding for this work?"	UNITED STATES - US
**Competing Interests**	The authors have declared that no competing interests exist.

Use the instructions below to enter a competing interest statement for this submission. On behalf of all authors, disclose any competing interests that could be perceived to bias this work—acknowledging all financial support and any other relevant financial or non-financial competing interests.<br><br>This statement is required for submission and **will appear in the published article** if the submission is accepted. Please make sure it is accurate and that any funding sources listed in your Funding Information later in the submission form are also declared in your Financial Disclosure statement.<br><br>View published research articles from *PLOS ONE* for specific examples.<br><br>**NO authors have competing interests**<br><br>Enter: *The authors have declared that no competing interests exist.*<br><br>**Authors with competing interests**<br><br>Enter competing interest details beginning with this statement:<br><br>*I have read the journal's policy and the authors of this manuscript have the following competing interests: [insert competing interests here]*<br><br>* typeset	
**Ethics Statement**<br><br>Enter an ethics statement for this submission. This statement is required if the study involved:<br><br>• Human participants<br>• Human specimens or tissue<br>• Vertebrate animals or cephalopods<br>• Vertebrate embryos or tissues	The Johns Hopkins University IRB approved this research (IRB00388076). Data were analyzed anonymously.

• Field research<br><br>Write "N/A" if the submission does not require an ethics statement.<br><br>General guidance is provided below. Consult the submission guidelines for detailed instructions. **Make sure that all information entered here is included in the Methods section of the manuscript.**	

**Format for specific study types**<br><br>**Human Subject Research (involving human participants and/or tissue)**<br>• Give the name of the institutional review board or ethics committee that approved the study<br>• Include the approval number and/or a statement indicating approval of this research<br>• Indicate the form of consent obtained (written/oral) or the reason that consent was not obtained (e.g. the data were analyzed anonymously)<br><br>**Animal Research (involving vertebrate animals, embryos or tissues)**<br>• Provide the name of the Institutional Animal Care and Use Committee (IACUC) or other relevant ethics board that reviewed the study protocol, and indicate whether they approved this research or granted a formal waiver of ethical approval<br>• Include an approval number if one was obtained<br>• If the study involved *non-human primates*, add *additional details* about animal welfare and steps taken to ameliorate suffering<br>• If anesthesia, euthanasia, or any kind of animal sacrifice is part of the study, include briefly which substances and/or methods were applied<br><br>**Field Research**<br><br>Include the following details if this study involves the collection of plant, animal, or other materials from a natural setting:<br>• Field permit number<br>• Name of the institution or relevant body that granted permission	
**Data Availability**<br><br>Authors are required to make the data underlying their research findings fully available, without restriction.<br><br>If the manuscript reports pilot or preliminary results, the data underlying those results must be made available at the time of publication. A Data Availability	N/A - No results are reported

Statement describing where the data can be found is required at submission. Your answers to this question constitute the Data Availability Statement and will be published in the article, if accepted. When legal or ethical requirements prohibit public data sharing, authors must indicate how researchers may obtain access to the data. See the PLOS Data Policy and FAQ for detailed information.<br><br>**Important:** Stating 'data available on request from the author' is not sufficient. If your data are only available upon request, select 'No' for the first question and explain your situation in the text box.<br><br>For protocols without pilot or preliminary data, authors are strongly encouraged to state how they plan to share research data from their study when it is completed or published.<br><br>If your study includes pilot data, will they be made fully available without restriction?	
**Describe where the data may be found in full sentences. If you are copying our sample text, replace any instances of XXX with the appropriate details.**<br><br>• If **no pilot data are reported** in the manuscript, please enter the following: *No datasets were generated or analysed during the current study. All relevant data from this study will be made available upon study completion.*<br>• If the manuscript reports pilot data **held in a public repository**, include URLs, accession numbers or DOIs. If this information will only be available after acceptance, indicate this by ticking the box below. For example: *All XXX files are available from the XXX database (accession number(s) XXX, XXX.).*<br>• If the manuscript reports pilot data contained **within the manuscript and/or**	All relevant data are within the manuscript and its Supporting Information files.

**Supporting Information files**, enter the following:<br>*All relevant data are within the manuscript and its Supporting Information files.*<br>• If neither of these applies but you are able to provide **details of access elsewhere**, with or without limitations, please do so. For example:<br><br>*Data cannot be shared publicly because of [XXX]. Data are available from the XXX Institutional Data Access / Ethics Committee (contact via XXX) for researchers who meet the criteria for access to confidential data.*<br><br>*The data underlying the results presented in the study are available from (include the name of the third party and contact information or URL).*<br>• This text is appropriate if the data are owned by a third party and authors do not have permission to share the data.<br><br>For study protocols with data management or sharing plans, authors are encouraged to briefly describe in their Data Availability Statement how data they generate will be made accessible when the study is completed. For example:<br>• Deidentified research data will be made publicly available when the study is completed and published.<br><br>* typeset	
Additional data availability information:	

Manuscript
Click here to access/download;Manuscript;17April2026 Design and Methods.docx

Study Protocol for First National Vasa Previa Perinatal Registry

Kayla E. Paulosky MD[1], Chiara Corbetta-Rastelli, MD[2], Efe Cudjoe, MD[3], David Esterquest, MD[4], Oluwaseun Fadairo, MD[5], Brittany Fickau, MD[6], Brenna Funfar, MD[7], Allison Perelman, MD[8], Naima Ross, MD[9], Antonio Santos-Roca, MD[11], Carrie Sibbald, MD[10], Logan Todhunter, MD[11], Bethany Waites, MD[12], Andrew Chon, MD[13], Chelsea A. DeBolt, MD[14], Kathryn Drennen, MD[11], Laura Hanks, MD[10], Angie Jelin, MD [1], Michelle Kush, MD [1], Tara Lynch, MD[5], Amol Malshe, MD[6], Lauren Miller, MD[12], Jessian Munoz, MD[15], PhD, Mary E. Norton, MD[2], Monica Rincon, MD[13], Ashley S. Roman, MD[9], Melissa Russo, MD[3], Rachelle St. Onge, MD[7], Yinka Oyelese, MD[17], Guy Steinberg, MD[16], Marika Toscano, MD [1]

Author Affiliations

1. Department of Gynecology & Obstetrics, Johns Hopkins Hospital, Baltimore, MD
2. Department of Obstetrics, Gynecology, and Reproductive Sciences, University of California San Francisco, San Francisco, CA
3. Department of Obstetrics and Gynecology, Women & Infants Hospital of Rhode Island, Providence, RI
4. Department of Obstetrics and Gynecology, Advocate Lutheran General, Park Ridge, IL
5. Department of Obstetrics and Gynecology, Albany Medical Center, Albany, NY
6. Department of Obstetrics and Gynecology, Cleveland Clinic Akron General, Akron, OH
7. Department of Obstetrics and Gynecology, University of Buffalo Jacobs School of Medicine, Buffalo, NY
8. Department of Obstetrics, Gynecology, and Reproductive Science, Icahn School of Medicine at Mount Sinai, New York, NY
9. Department of Obstetrics and Gynecology, NYU Grossman School of Medicine, New York, NY

10. Department of Obstetrics and Gynecology, University of Wisconsin School of Medicine and Public Health, Madison, WI
11. Department of Obstetrics and Gynecology, University of Rochester Medical Center, Rochester, NY
12. Department of Obstetrics and Gynecology, University of Colorado Anschutz School of Medicine, Aurora, CO
13. Department of Obstetrics and Gynecology, Oregon Health and Science University, Portland, OR
14. Department of Obstetrics, Gynecology, and Reproductive Science, Icahn School of Medicine at Mount Siani, New York, NY
15. Department of Obstetrics and Gynecology, Baylor College of Medicine, Houston, TX
16. Department of Obstetrics and Gynecology, Advocate Aurora Institute, Downers Grove, IL
17. Department of Obstetrics and Gynecology, Brigham and Women's Hospital, Boston, MA

*Corresponding author

Email: Mtoscan3@jh.edu (MT)

Abstract

Vasa previa is a rare but potentially catastrophic obstetric condition characterized by unprotected fetal blood vessels over or adjacent to the internal cervical os. Although advances in prenatal ultrasound have dramatically improved neonatal survival through planned cesarean delivery, many aspects of vasa previa remain poorly understood. Existing studies are limited by small sample sizes, single-center designs, heterogeneous definitions, and variable reporting of maternal, neonatal, and health system outcomes. As a result, uncertainty persists regarding the natural history of vasa previa, optimal antenatal management strategies, patient experience, and resource utilization. Here we describe, to our knowledge, the first United States multicenter registry of pregnancies complicated by vasa previa without concurrent placenta previa, the U.S. Vasa Previa Registry (US-VPR), established to improve scientific understanding of the clinical characteristics, management, and outcomes of pregnancies complicated by vasa previa. This report describes the rationale, design, and implementation of a large, multicenter, retrospective registry focused on characterizing demographics, risk factors, placental pathology, natural history, antenatal management strategies, maternal outcomes, neonatal outcomes, and healthcare utilization associated with vasa previa. The US-VPR includes contributions from 17 institutions and represents the largest multicenter cohort of pregnancies complicated by vasa previa reported to date. The registry captures a substantially larger and more diverse population than previously reported single-center cohorts and provides an opportunity to evaluate variation in management practices and associated outcomes. By leveraging multicenter collaboration and standardized data collection, this registry offers high-quality, real-world evidence to inform clinical decision-making, guide individualized management strategies, improve patient counseling, and optimize maternal and neonatal outcomes.

.

Introduction

Vasa previa is a rare but potentially catastrophic obstetric condition affecting ~1 in 1200 pregnancies.[1] It is defined by fetal blood vessels that traverse the amniotic membranes over or adjacent to the internal cervical os.[2] Because these vessels are unsupported by placental tissue or the umbilical cord, they are vulnerable to compression or rupture during labor or membrane rupture, which can result in rapid fetal exsanguination and acute fetal compromise.[3] Historically, undiagnosed vasa previa was associated with perinatal mortality rates exceeding 40%; however, advances in prenatal ultrasound diagnosis followed by planned prelabor cesarean delivery have improved neonatal survival to nearly 100%.[4-12]

Approximately 80% of cases of vasa previa are associated with one or more identifiable risk factors.[13,14] These include velamentous cord insertion, second-trimester low-lying placenta or placenta previa, conception via assisted reproductive technologies, bilobed or succenturiate placentas located in the lower uterine segment, and multiple gestations.[11,14-18]

Prenatal ultrasound has demonstrated high sensitivity for detection of vasa previa.[5,19,20] Known risk factors allow for risk stratification during the second-trimester anatomy ultrasound, which is the gestational window when vasa previa is most commonly diagnosed.[21] Screening for vasa previa as a part of standardized assessment at the second-trimester anatomy ultrasound is recommended, utilizing transvaginal ultrasound for confirmation.[3,22] While many institutions employ risk-based screening strategies, a recent international expert consensus published in 2024 recommends universal screening at the time of the second-trimester anatomy scan to improve early detection and outcomes.[23,24]

Vasa previa is commonly categorized into three subtypes based on underlying placental and cord anatomy. Type I, the most common subtype, is characterized by velamentous cord

insertion with unprotected vessels crossing the internal os. Type II occurs when fetal vessels traverse the membranes between a bilobed or succenturiate placenta. Type III describes aberrant fetal vessels coursing through the membranes over the cervical os that do not meet criteria for Type I or II. Differences in risk profiles, clinical behavior, and outcomes between these subtypes remain incompletely characterized.[25-27]

Despite advances in diagnosis, important controversies persist regarding the definition and management of vasa previa. There is current uncertainty regarding the threshold for how close fetal vessels must be to the internal cervical os to meet diagnostic criteria. A distance of 2 cm has been widely adopted; however, this strict cutoff has been increasingly questioned. Emerging evidence suggests that fetal vessels located within 2–5 cm of the internal os, often termed “low-lying fetal vessels,” may carry risks similar to classic vasa previa, challenging the clinical utility of a rigid distance-based definition. [28-32]

Similarly, the natural history of vasa previa across gestation remains poorly defined. Reported rates of spontaneous resolution range from approximately 6% to 39%, with higher rates observed when diagnosis occurs earlier in gestation.[2,9,10,33,34] These estimates are limited by small sample sizes, heterogeneous imaging protocols, and inconsistent definitions of resolution. Data identifying predictors of preterm delivery or persistent disease remain limited.[33]

Although prenatal detection has markedly improved survival, antenatal management strategies for vasa previa vary widely across institutions.[35,36] Contemporary reviews and professional society guidance acknowledge limited evidence to inform decisions regarding surveillance intensity, inpatient versus outpatient management, and optimal timing of delivery.[37,38] Recent cohort studies demonstrate excellent survival among prenatally diagnosed cases but continue to reveal substantial heterogeneity in management approaches and resource

utilization.[39-41] In parallel, an international Delphi survey of 68 experts reached consensus on several aspects of definition, screening, and clinical management; however, the evidence base informing these recommendations remains constrained by small cohorts and meta-analyses with inherent limitations.[42]

Fetoscopic laser photocoagulation has emerged as an investigational treatment option for select cases of vasa previa, with case reports and small series describing successful procedures.[43-49] However, this approach remains highly specialized and is not considered standard of care.

In addition to management variability, neonatal outcomes associated with vasa previa have been incompletely described. Prior studies and meta-analyses have focused primarily on gestational age at delivery, birthweight, Apgar scores, neonatal anemia, transfusion requirements, and mortality. [12,37,50] Less is known about long-term postnatal clinical courses, neonatal intensive care unit utilization, and broader health system impacts. Concurrently, recent epidemiologic data suggest that vasa previa may be more common than previously recognized, underscoring the need for improved understanding of its clinical and resource implications in contemporary practice.[1,20]

Despite excellent neonatal survival with prenatal diagnosis, pregnancies complicated by vasa previa are associated with substantial care burden and psychosocial impact for patients.[11] Population-based and cohort studies have shown that affected patients frequently undergo intensive antenatal surveillance, activity restriction, and prolonged hospitalization.[12,51] Themes emerge about receiving inconsistent information about the condition.[51] These findings underscore that vasa previa management extends beyond prevention of perinatal mortality and highlight the need to better characterize patient experience and resource utilization alongside traditional perinatal outcomes.[52] To address these persistent gaps, we established a multicenter

registry of pregnancies complicated by vasa previa without concurrent placenta previa. This registry currently includes 17 institutions of varying sizes across the United States, enhancing generalizability and enabling capture of rare outcomes. Prior to this effort, the largest single cohort reporting granular clinical details of vasa previa included 155 patients.[19] We aim to comprehensively characterize patient demographics, risk factors, placental pathology, natural history, recurrence risk, antenatal management strategies, neonatal outcomes, and maternal outcomes associated with vasa previa.[37,52] As clinical management evolves toward more individualized and risk-adapted approaches, these data will inform evidence-based decision-making, optimize patient counseling, and improve outcomes for this rare but high-risk obstetric condition.

Methods

Rationale for a Registry

Over the past decade, patient registries have become increasingly common across medicine because they are uniquely positioned to address important questions that cannot be adequately answered by randomized controlled trials (RCTs) or small observational studies alone.

Although RCTs remain the gold standard for evaluating the efficacy of specific interventions, they are poorly suited to the study of rare obstetric conditions such as vasa previa. The low incidence of vasa previa makes prospective randomized studies impractical, and existing cohorts are typically limited by small sample sizes, single-center designs, and heterogeneous inclusion criteria. Moreover, RCTs are not designed to characterize the natural

history of disease, evaluate variation in real-world clinical management, assess patient experience, or quantify healthcare utilization and system-level burden. As a result, many critical aspects of vasa previa—including risk stratification, antenatal management strategies, timing of delivery, maternal outcomes, neonatal morbidity, and resource use—remain incompletely understood.

Patient registries are designed to capture large volumes of data generated during routine clinical practice without altering clinical care. By enrolling patients across diverse institutions and practice settings, registries enable comprehensive characterization of disease epidemiology, clinical heterogeneity, and real-world outcomes. For rare conditions, registries provide sufficient sample size to study uncommon presentations, management strategies, and adverse events that no single institution encounters frequently enough to evaluate independently. In addition, registries allow for the assessment of variation in practice patterns, identification of best practices, and evaluation of healthcare utilization and costs—areas that are largely inaccessible through traditional clinical trials.

For vasa previa specifically, a national registry offers an opportunity to address persistent gaps in the literature. Baseline registry data can provide detailed information on patient demographics, obstetric history, placental and cord abnormalities, imaging findings, and risk factors associated with vasa previa. Longitudinal follow-up can elucidate the natural history of the condition, rates of persistence or apparent resolution, recurrence risk, and real-world antenatal management strategies, including inpatient versus outpatient care and timing of delivery. Importantly, a registry can also capture maternal outcomes, neonatal outcomes beyond survival alone, and healthcare utilization such as hospital admissions, length of stay, and neonatal intensive care unit use—outcomes that remain underreported in existing studies.

By leveraging multicenter collaboration and standardized data collection across institutions, the US-VPR is designed to provide comprehensive, real-world evidence on pregnancies complicated by vasa previa. Findings from this registry will support evidence-based clinical decision-making, inform individualized management strategies, improve patient counseling, and ultimately contribute to optimizing maternal and neonatal outcomes for this rare but high-risk obstetric condition.

Initial Implementation

Registry design and setting

This study was designed as a multicenter, retrospective, observational perinatal registry–style cohort of pregnancies complicated by vasa previa without concurrent placenta previa from January 1, 2011, through May 1, 2023. To date, the registry includes 17 U.S. medical centers (**Table 1**), each with established maternal–fetal medicine divisions and advanced obstetric imaging capabilities. Participating sites include tertiary and quaternary referral centers that care for both routine and high-risk obstetric populations. Although the majority of sites are academic institutions, several serve broad regional catchment areas and function as safety-net providers for medically complex pregnancies.

At the time of manuscript submission, the registry includes 17 participating institutions. Data collection has been completed at 12 sites, with continued accrual ongoing at 3 sites and anticipated to be completed by July 31, 2026. Data analysis has not yet begun and is expected to commence on May 1, 2026, with initial results anticipated by September 1, 2026.

Participating institutions are geographically distributed across the Northeast, Midwest, West Coast, and Southern United States, supporting inclusion of a diverse patient population and a range of practice patterns (**Figure 1**).

Recruitment of centers occurred through professional networks in obstetrics and maternal–fetal medicine, direct outreach to institutions with expertise in high-risk pregnancy care, and through review of presentations at national and regional scientific meetings. Informal professional referral and word-of-mouth also contributed to recruitment. Institutional participation requirements are displayed in **Table 2.**

This initial formation of the US-VPR registry is retrospective in nature, capturing historical cases identified through existing obstetric ultrasound databases, electronic health records, and billing systems. No interventions were introduced as part of the study; all care was delivered per local clinical practice, rendering the registry purely observational.

The registry is health-system–based, drawing from large academic and tertiary referral centers with specialized maternal–fetal medicine ultrasound services rather than from population-level surveillance systems. This design was selected to enable detailed phenotyping, imaging review, placental pathology assessment, and granular neonatal outcome ascertainment for a rare obstetric diagnosis.

Johns Hopkins University serves as the central coordinating and data management center for the registry. Oversight was provided by the study principal investigator in collaboration with designated site principal investigators at each participating institution. A central governance structure was implemented to promote data consistency and scientific rigor. This included: (1) Standardized inclusion and exclusion criteria; (2) Uniform variable definitions and data dictionaries; (3) Centralized data storage in a secure REDCap database housed at the coordinating center.

Each participating institution retained responsibility for local regulatory oversight, including Institutional Review Board (IRB) approval and protection of protected health

information, and data use agreements were executed between institutions prior to data sharing. During the process of case identification and chart abstraction at participating institutions, site investigators had access to identifiable patient information within their local electronic health records. Only de-identified data were subsequently transferred to the coordinating center for analysis, and the study team at the coordinating site did not have access to direct patient identifiers. Data access was restricted to IRB-approved study personnel. Requests for use of the pooled, multicenter dataset for secondary analyses require written approval and, when applicable, submission of protocol amendments to the coordinating center's IRB.

Policies governing data use, analysis, and publication emphasized collaborative authorship, transparency, and scientific integrity. The principal investigator convened regular investigator meetings to review data quality and coordinate analysis and dissemination of findings.

Study population

The US-VPR aims to capture data on pregnancies complicated by vasa previa across participating centers. As with all observational registries, some degree of unavoidable selection exists. First, inclusion is limited to pregnancies managed at institutions that have elected to participate in the registry, obtained institutional review board (IRB) approval (or participated through an approved reliance agreement), and executed the required data use for collaboration agreement. Second, cases must be identified and diagnosed as vasa previa by the treating clinical team based on predefined diagnostic criteria. Third, data inclusion depends on successful case ascertainment and abstraction by local study personnel, as data entry is voluntary rather than mandatory at participating sites.

Beyond these practical considerations, the registry includes pregnancies with prenatal ultrasound diagnosis of vasa previa (fetal vessels ≤2 cm from the internal cervical os) or low-lying fetal vessels (>2–5 cm from the internal cervical os), delivery at a viable gestational age, and availability of complete maternal delivery and corresponding neonatal records. Pregnancies are eligible regardless of maternal age, race or ethnicity, insurance status, comorbidities, plurality, or treatment setting. Both singleton and multiple gestation pregnancies are eligible for inclusion. Pregnancies complicated by concurrent placenta previa are excluded, consistent with the registry's design and analytic focus. Individuals with resolved vasa previa were included but will be analyzed as a separate cohort. Inclusion and exclusion criteria are summarized in **Table 3**.

Data are collected using a standardized electronic data capture system and are abstracted retrospectively from the medical record. All data collection instruments are administered in English; however, data abstraction is performed by trained site personnel using the medical record, and no direct patient-facing questionnaires were required for inclusion in the present cohort.

Population Representativeness

This registry represents a health-system–based cohort drawn from large academic and tertiary referral centers with specialized maternal–fetal medicine ultrasound services. As such, the study population reflects patients receiving care for both routine and high-risk pregnancies, including referrals for suspected or confirmed vasa previa.

Although the cohort is not population-based, the multicenter design and broad geographic distribution of participating institutions enhance heterogeneity in patient demographics and clinical practice patterns. This design prioritizes depth of clinical phenotyping and outcome ascertainment for a rare obstetric condition over population-level incidence estimation.

Formal comparison to underlying source populations at each site was not performed; however, inclusion of all eligible cases identified during the study period at participating institutions minimizes selection bias within health systems.

Data sources

Primary Data Sources

The primary data source for this registry is the electronic health record (EHR) at each participating institution. Clinical data are derived from maternal and neonatal EHRs, including prenatal, delivery, and postpartum encounters.

EHR data are supplemented by institutional obstetric ultrasound databases, which served as the primary source for case identification and detailed characterization of vasa previa, cord insertion, placental morphology, and vessel location. These systems included structured ultrasound reporting platforms routinely used in maternal–fetal medicine practice.

To ensure comprehensive and accurate variable capture, manual chart abstraction is performed by clinically trained study personnel at each site. Data are entered into a registry-specific REDCap database using standardized variable definitions and data dictionaries developed by the coordinating center.

Data Capture Frequency and Timepoints

Data collection for this initial registry creation has thus far been retrospective and episodic, rather than real-time. Each participating site performed a one-time identification and abstraction of all eligible cases occurring during the study period.

Clinical data are captured across key obstetric and neonatal timepoints, including:

- Antepartum: prenatal diagnosis, imaging findings, antenatal surveillance, inpatient versus outpatient management, and complications
- Delivery: gestational age, mode and timing of delivery, delivery indications, and operative findings
- Postpartum and neonatal period: immediate neonatal outcomes, neonatal intensive care unit (NICU) admission and course, and discharge-related variables

No prospective follow-up or repeated data extraction was performed beyond what is available in the existing medical record for the index pregnancy and neonatal hospitalization.

Participant identification

Pregnancies complicated by vasa previa included in the US-VPR were identified using a multi-step, confirmatory case ascertainment approach at each participating institution (**Supplemental Table 2**).

Initial case identification was performed through:

- Queries of institutional obstetric ultrasound databases used in routine maternal–fetal medicine practice, which contain structured documentation of placental location, cord insertion, and fetal vessel anatomy.
- Queries of hospital administrative and billing systems for ICD codes diagnoses associated with vasa previa.

These initial electronic queries were intentionally inclusive and were followed by manual chart review to confirm eligibility. A pregnancy was classified as affected by vasa previa or low-lying fetal vessels only if the diagnosis was documented on prenatal ultrasound imaging and confirmed by trained clinician-performed chart review. Administrative diagnosis codes (e.g., ICD-10) were used solely to aid case finding and were not relied upon in isolation to define exposure status. Final case adjudication was based on ultrasound findings and clinical documentation rather than billing codes alone.

Dating of Pregnancy

Gestational dating was derived from the best obstetric estimate documented in the medical record, consistent with standard clinical practice at participating institutions. Pregnancy start date was determined using the estimated gestational age assigned by the treating clinicians. Pregnancy end date was defined as the date of delivery. Pregnancies ending in fetal demise were dated based on the documented delivery or diagnosis date. Gestational age at key timepoints—including diagnosis, initiation of inpatient management, and delivery—was calculated using the recorded gestational age corresponding to those clinical encounters.

Handling of Complex Scenarios

Several potential complexities inherent to registry-based pregnancy identification are addressed as follows:

- Early pregnancy loss: Pregnancies that did not progress to delivery at a viable gestational age are excluded by design, as eligibility required delivery at a viable gestational age with complete delivery records available.

- Stillbirth: Pregnancies complicated by stillbirth at or beyond viability were eligible for inclusion for ultrasound and antenatal management variables. Delivery and neonatal outcome variables may be collected, but will be excluded from analyses regarding delivery timing, mode of delivery, and neonatal outcomes.
- Transfers between facilities: For patients transferred between institutions, pregnancy episodes are attributed to the site responsible for delivery, with antenatal data abstracted from available records within the delivering institution's EHR.
- Overlapping or conflicting records: In cases of discrepant documentation (e.g., differing descriptions of vessel location or placental morphology), ultrasound reports and specialist maternal–fetal medicine documentation are prioritized over administrative data. Ambiguous cases are resolved through chart review and individual image review by site investigators.

Each eligible pregnancy is treated as a distinct analytic episode, even when individuals contributed more than one pregnancy during the study period. This approach reflects the pregnancy-specific nature of vasa previa risk, diagnosis, and management.

Variables of interest

The registry captures data across five core domains: maternal demographics, medical and obstetric history, pregnancy complications, delivery characteristics, and neonatal outcomes which were informed by prior publications.[52,53] Maternal demographic variables include age, race, ethnicity, and body mass index. Medical and obstetric history include gravidity, parity, prior uterine surgery, and use of assisted reproductive technologies. Pregnancy complications and

antenatal course include placental and cord characteristics, vasa previa subtype, antenatal management strategies, and antepartum bleeding events. Delivery characteristics include gestational age at delivery, mode and timing of delivery, and delivery indications. Neonatal outcomes include birthweight, Apgar scores, need for resuscitation or transfusion, NICU admission and length of stay, and major neonatal morbidities.

Outcome measures are defined using standardized, clinically accepted operational definitions where available (**Supplemental Table 1**). Severe maternal morbidity is classified using Centers for Disease Control and Prevention (CDC) indicators. Neonatal outcomes are abstracted directly from the medical record using established clinical definitions. When applicable, outcome definitions were informed by prior studies and consensus guidelines.

Covariates include clinical factors (e.g., gestational age at diagnosis, plurality, cord insertion type), social determinants of health available in the medical record (e.g., race and ethnicity), and health system variables such as inpatient versus outpatient management and site of care. These variables were selected a priori based on biologic plausibility and relevance to antenatal management and neonatal outcomes in pregnancies affected by vasa previa.

A complete variable list, including variable definitions, is provided in **Supplemental Table 1**.

Data quality assurance

Data collection is standardized across participating sites using a centralized data dictionary and prespecified variable definitions developed by the coordinating center. All data are entered into a registry-specific REDCap database, which served as the common data structure

for the study. Prior to data entry, site investigators received guidance on variable definitions, inclusion criteria, and abstraction procedures to promote cross-site consistency.

Cross-site harmonization is supported through ongoing communication between the coordinating center and participating institutions, including regular investigator meetings to review data collection practices, clarify definitions, and address discrepancies identified during abstraction.

Data quality is assessed using a combination of automated and manual checks. Built-in REDCap functionality is used to implement range checks (e.g., gestational age, birthweight) and logic checks (e.g., delivery date occurring after diagnosis date; gestational age consistency across timepoints). Fields critical to primary study objectives are designated as required where feasible to minimize missing data.

Patterns of missingness are monitored at the site level, and sites are contacted to resolve missing or implausible values when identified. No formal missingness thresholds are used to exclude sites or cases; instead, missing data will be addressed descriptively in analysis.

All cases included in the registry undergo manual chart review to confirm eligibility and abstract key variables. For pregnancy identification, concordance between administrative data, ultrasound reports, and clinical documentation was assessed during chart review, with prenatal ultrasound findings prioritized for confirmation of vasa previa diagnosis and classification.

Administrative diagnosis codes are used solely for case-finding and are not relied upon to define exposure status. As such, formal algorithm validation metrics are not calculated. Ultrasound-based diagnostic criteria and outcome definitions were informed by prior literature and established clinical practice guidelines.

Ethical considerations

This study was reviewed and approved by the Institutional Review Board at the original site, which served as the coordinating IRB for the multicenter study. Participating institutions obtained local IRB approval in accordance with their institutional policies.

Given the retrospective design and exclusive use of existing clinical data, the registry is conducted with a waiver of informed consent and a waiver of HIPAA authorization. The waivers were granted on the basis that the research posed no more than minimal risk to participants, could not practicably be conducted without access to protected health information, and involved no direct contact with participants.

Statistical analysis and Timeline

This report is primarily descriptive and focuses on the design of the registry and baseline characteristics of the included institutions. The initial set of investigators of the US-VPR has identified several planned analyses using the data collected. The initial publication will focus on describing the baseline characteristics of the registry cohort, including patient demographics, obstetric history, placental and cord abnormalities, imaging findings, and antenatal management practices. Subsequent analyses will examine clinically relevant subgroups, including comparisons by vasa previa subtype (Types I, II, and III), distance of fetal vessels from the internal cervical os, and inpatient versus outpatient management strategies.

Planned analyses will also evaluate differences in maternal outcomes, neonatal outcomes, and healthcare utilization across these subgroups, with the goal of identifying patterns that may inform risk stratification and individualized clinical management. Together, these studies aim to clarify heterogeneity in presentation, management, and outcomes among pregnancies

complicated by vasa previa and to provide an evidence base to support more tailored counseling and care.

Handling of Missing Data

Given the retrospective design, missing data are anticipated for certain variables. Analyses will be conducted using a complete-case approach for variables of interest, with the extent of missingness reported descriptively. No formal imputation procedures were planned, as the primary intent of the registry was characterization rather than causal inference.

Discussion

Strengths and limitations of the registry

This registry represents one of the largest multicenter cohorts of pregnancies complicated by vasa previa without concurrent placenta previa, a rare obstetric condition that has historically been limited to small case series. The multicenter design, inclusion of geographically diverse tertiary referral centers, and extended study period enhance the breadth of clinical practice patterns and outcome ascertainment.

A key strength of the registry is the use of prenatal ultrasound confirmation for case adjudication, rather than reliance on administrative codes alone, improving diagnostic accuracy. Additionally, all cases meeting criteria underwent a multilevel adjudication process, first by the individual institutions and then validated by the primary site. The registry includes granular antenatal management data, detailed placental pathology findings, and comprehensive neonatal outcomes, enabling characterization of both clinical care processes and short-term neonatal morbidity.

Standardized data collection procedures, centralized data management, and manual chart review across sites further support data quality. The pregnancy-level analytic framework allows evaluation of natural history, management strategies, and recurrence risk, which are particularly relevant for counseling and clinical decision-making in vasa previa.

Several limitations should be considered when interpreting findings from this registry. The retrospective design introduces the potential for missing data, misclassification, and documentation variability across sites. While all sites had the same inclusion and exclusion criteria, there could be local variances in how cases were collected and met criteria. Management strategies were not standardized and reflect local practice patterns, limiting causal inference regarding comparative effectiveness of specific antenatal approaches. Additionally, sample sizes for comparative analyses may be smaller, limiting power.

The registry is health-system–based and largely composed of academic referral centers, which may limit generalizability to lower-resource or non-referral settings. Population-level incidence estimates could not be derived, and formal comparisons to underlying source populations were not performed.

Long-term maternal and child outcomes beyond the neonatal hospitalization are not consistently available, and patient-reported outcomes are not captured. Finally, although recurrence risk will be assessed, follow-up is limited to subsequent pregnancies documented within participating health systems.

Data access

Participating sites retain the right to independently analyze, present, and publish their own internal data without the approval of the US-VPR research group, on the condition that they acknowledge the US-VPR project's role in the research design, data collection and project

management. Secondary statistical analyses of the entire US-VPR database may also be proposed by any participating site who has contributed a minimum number of baseline participants to the registry. Investigators may request access to registry data for secondary analyses through a formal data access request process. Proposals are reviewed by the registry steering committee to ensure scientific merit, feasibility, and alignment with registry priorities, as well as to avoid overlap with planned or ongoing analyses. Approved projects will be conducted in accordance with registry governance, data use agreements, and authorship policies.

Upcoming Developments

The US-VPR was designed as a foundational platform with the capacity to evolve over time. A key upcoming development is a planned transition from a static, retrospective registry to a living registry with ongoing data accrual. This evolution will allow the registry ongoing capture of contemporary practice patterns, longitudinal outcomes, and changes in management strategies as evidence and clinical guidance continue to evolve.

Author Contributions

Kayla Paulosky contributed to conceptualization, investigation, formal analysis, writing-original draft preparation, **Marika Toscano** contributed to conceptualization, data curation, formal analysis, methodology, project administration, supervision, validation, and writing-original draft preparation. All other included authors contributed to investigation, data curation, oversite at their respective institutions, and writing-review and editing. **All** authors approved the final submitted manuscript.

503

Table 1. Participating Institutions in the Vasa Previa Multicenter Registry

**Name**	**Location**	**Number of participants**
Advocate Aurora Health (now Advocate Health)	Multiple sites, IL & WI	41
Albany Medical Center	Albany, NY	14
Baylor College of Medicine	Houston, TX	Ongoing data collection
Brigham and Womens Hospital	Boston, MA	Ongoing data collection
Warren Alpert Medical School of Brown University<br>Women & Infants Hospital of Rhode Island	Providence, RI	Ongoing data collection
University at Buffalo (State University of New York)	Buffalo, NY	24
Cleveland Clinic	Cleveland, OH	81
University of Colorado Anschutz Medical Campus	Aurora, CO	68
Oregon Health & Science University	Portland, OR	28
University of Rochester Medical Center	Rochester, NY	65

Icahn School of Medicine at Mount Sinai	New York, NY	68
University of California, San Francisco	San Francisco, CA	112
New York University Grossman School of Medicine	New York, NY	97
University of Wisconsin School of Medicine and Public Health	Madison, WI	47
Johns Hopkins University School of Medicine	Baltimore, MD	95

Table 2. Institution participation requirements

Institution Participation Requirements
1. Elected to participate in registry voluntarily<br>2. Obtained institutional review board approval at respective institution<br>3. Completed required data use and collaboration agreement<br>4. Completed case ascertainment at institution through methods determined by local study personnel<br>5. Completed and validated electronic medical record data extraction

Table 3. Eligibility for the vasa previa registry

Inclusion Criteria	Exclusion Criteria
1. Pregnancies managed at participating institutions with IRB approval and data use agreements<br>2. Prenatal ultrasound diagnosis of vasa previa (fetal vessels ≤2 cm from the internal cervical os) or low-lying fetal vessels (>2–5 cm from the internal cervical os)<br>3. Delivery at viable gestational age<br>4. Live born or stillborn infants<br>5. Complete maternal delivery and neonatal records available<br>6. Singleton or multiple gestation pregnancies<br>7. All maternal demographics and clinical characteristics eligible (no restrictions based on age, race/ethnicity, insurance status, or comorbidities)<br>8. Resolved vasa previa included but analyzed as a separate cohort	1. Concurrent placenta previa<br>2. Pregnancies without ultrasound records, delivery records or neonatal records

Supplemental Table 1. Data Elements Collected in the Vasa Previa Without Concurrent Placenta Previa Multicenter Registry. This table summarizes all data elements collected in the multicenter registry. Variables collected per fetus (Fetus A, B, and C in multiple gestations) use identical definitions and coding unless otherwise specified. Free-text variables are noted but not exhaustively itemized. Coding reflects the original REDCap instrument.

**Demographics**
Maternal age at delivery
Height
Weight
Race
Ethnicity
Gravidity and Parity
**Maternal Medical History**
Pregnancy conceived through assisted reproductive technology (ART). If yes, type of ART
Prior C-section? If yes, number
Prior myomectomy?
Prior Uterine septum resection?
Prior cervical surgery?
Other uterine instrumentation?
**Site information**
How many L&D units are included at your site where patients with vasa previa are admitted/delivered?
Level of maternal care per L&D unit
Level of neonatal care per L&D unit
Is there 24/7 resident coverage of unit, per unit
Is your antepartum unit located on the same floor of the hospital as your L&D unit, per L&D unit
Describe the logistics of moving patient from antepartum to L&D for urgent delivery
**Ultrasound Data**
Number of gestations
Type of ultrasound machine used
Type of ultrasound transducer used (if known)
Type of ultrasound done (viability, dating, nuchal translucency, anatomic, follow-up anatomy, follow-up growth, other)
Was a transvaginal ultrasound done?
Gestational age at time of first ultrasound exam showing vasa previa
Type of vasa previa found on ultrasound (Type 1, Type 2, Type 3, low-lying fetal vessels, type not documented, other)

What vessel(s) was involved in the vasa previa (select all which apply). Umbilical artery, umbilical arteries, umbilical vein, unknown)
What was the diameter (mm) of each vessel involved in the vasa previa (if measured/known)
Placental location (anterior, posterior, lateral, fundal, other)
Was an accessory placental lobe present? If so, which type (succenturiate, bilobed, other)
Where was the location of the accessory placental lobe?
Type of umbilical cord insertion into the placenta (normal, marginal, velamentous, other)
Is placenta previa or low-lying placental present on the current ultrasound?
Distance from placental edge to internal cervical os
Distance between fetal vessels and internal cervical os (if measured)
Was cervical length measurement performed?
Cervical length measurement
Was pulsed Doppler assessment for the fetal vessel performed?
Additional ultrasound done?
Did vasa previa resolve on subsequent ultrasound exams?
How was resolution of the vasa previa defined? Please describe
Was a procedure performed on this vasa previa (ie. Laser coagulation)? If so, describe.
Gestational age at vasa previa resolution (weeks and days gestation)
**Antenatal management**
Was patient admitted during the antenatal period for any reason during their pregnancy?
How many hospital antepartum admissions did the patient have total?
What were the reasons for hospital antenatal admission during their pregnancy?
Gestational age at hospital admission
Where was the patient admitted? (L&D unit, antepartum unit, intensive care unit, other)
Type of antenatal testing
How often did the patient get non-stress tests during admission?
Describe NST frequency if you selected “other”
How often did the patient get a biophysical profile during admission?
Describe biophysical profile frequency if you selected “other”
Describe other antenatal testing that was done
How often did the patient get growth ultrasound during admission?
Did the patient have any of the following pregnancy-related complications?
How many bleeding episodes did the patient have during the pregnancy after the vasa previa was diagnosed?
Describe any other pertinent aspects of the patient’s antenatal hospitalization
**Delivery**
Gestational age
Mode of delivery
Was this an emergent delivery
What was the indication for delivery (preterm labor, preterm prelabor rupture of membranes, vaginal bleeding, non-reassuring fetal heart rate tracing)
How many doses/days of antenatal corticosteroids did the patient receive prior to delivery?

Was magnesium sulfate administered for fetal neuroprotection prior to delivery?
Did the patient receive tocolytics?
Did operative notes or delivery summary remark on gross examination of placenta and cord, were they consistent with vasa previa?
Placental pathology report
Did the patient have a hysterectomy at the time of delivery?
Was there a maternal death?
Was there severe maternal morbidity (as defined by CDC)
What was the overall maternal length of stay (# days) in the hospital during this admission
Did the patient have an Edinburgh Perinatal Depression Scale done at the 6–week postpartum appointment? Is yes, score
Did the patient get referred to behavioral health in the postpartum period at or before 6-week postpartum visit?
Is it documented in the patient's postpartum notes that they suffered from traumatic birth or post-traumatic stress disorder. Acute stress reactions?
Do postpartum notes document any impact of hospitalization or delivery on the patient's life?
**Neonatal Outcomes (per fetus)**
Was there a fetal death in utero diagnosed for this fetus?
Gestational age at fetal demise diagnosis
Pertinent details related to etiology of fetal demise in utero (if known) based on placental pathology autopsy
Neonatal birthweight in grams
Apgar score at 1 and 5 minutes
Umbilical cord blood gas drawn?
Please input the umbilical cord blood pH here
Umbilical cord blood gas origin (arterial preferred
Where was the newborn admitted after birth?
Was the newborn later transferred to the NICU?
Day of life when NICU transfer occurred
Reason for NICU transfer
Was the newborn hemoglobin level drawn within the first 48 hours of life? If so, what is the level
Did the neonate require a blood transfusion within 72 hours of birth?
Did the neonate require assisted ventilation immediately following delivery? (within 0 to 6 hours of birth)
What was the newborn's disposition after NICU admission?
Please describe circumstances of neonatal death
What was the total number of days the newborn spent in an intensive care unit after birth?
What is the total number of days the newborn spent in the hospital, from day of delivery to the day of discharge?
Did the neonate have radiographic evidence of an intraventricular hemorrhage (IVH)? If yes, IVH grade

Did the neonate develop a diagnosis of bronchopulmonary dysplasia (BPD)? If yes, what was the severity
Was the neonate diagnosed with necrotizing enterocolitis? If yes what stage
Was the newborn discharge on respiratory support?
Was the newborn discharged on feeding support?
Please discuss any other pertinent complications occurring during the newborn period
**Recurrence**
Did the patient have another pregnancy?
Did this patient have recurrent vasa previa in subsequent pregnancy?

Supplemental Table 2. Multistep Case Ascertainment Strategy for Identification of Vasa Previa

Step	Data Source	Ascertainment Method	Purpose / Rationale
**1**	Electronic medical record (EMR)	Identification of potential cases using diagnosis codes, problem lists, and ultrasound report keywords related to vasa previa	To capture all possible vasa previa cases with high sensitivity
**2**	Prenatal ultrasound reports	Review of second- and third-trimester ultrasound reports documenting suspected or confirmed vasa previa, low-lying fetal vessels, velamentous cord insertion, or abnormal placental morphology	To identify cases based on imaging findings rather than diagnosis codes alone
**3**	Maternal–fetal medicine (MFM) consultation notes	Manual chart review of specialist notes describing suspected or confirmed vasa previa and management plans	To corroborate imaging findings and clinical diagnosis
**4**	Delivery records and operative reports	Review of cesarean delivery reports for intraoperative confirmation of vasa previa or low-lying fetal vessels	To confirm diagnosis at delivery when available
**5**	Placental pathology reports	Review of placental examination documenting velamentous cord insertion, fetal vessels within membranes, or placental anomalies consistent with vasa previa	To provide anatomic confirmation and reduce misclassification
**6**	Local investigator adjudication	Independent confirmation by site investigators using predefined diagnostic criteria	To ensure consistency of case definition across institutions
**7**	Central study team review	Secondary review of submitted cases with resolution of discrepancies through consensus	To standardize final case inclusion across sites

Figure Click here to access/download;Figure;Figure 1.docx

Figure 1. Geographic Distribution of Participating Institutions in the Vasa Previa Multicenter Registry

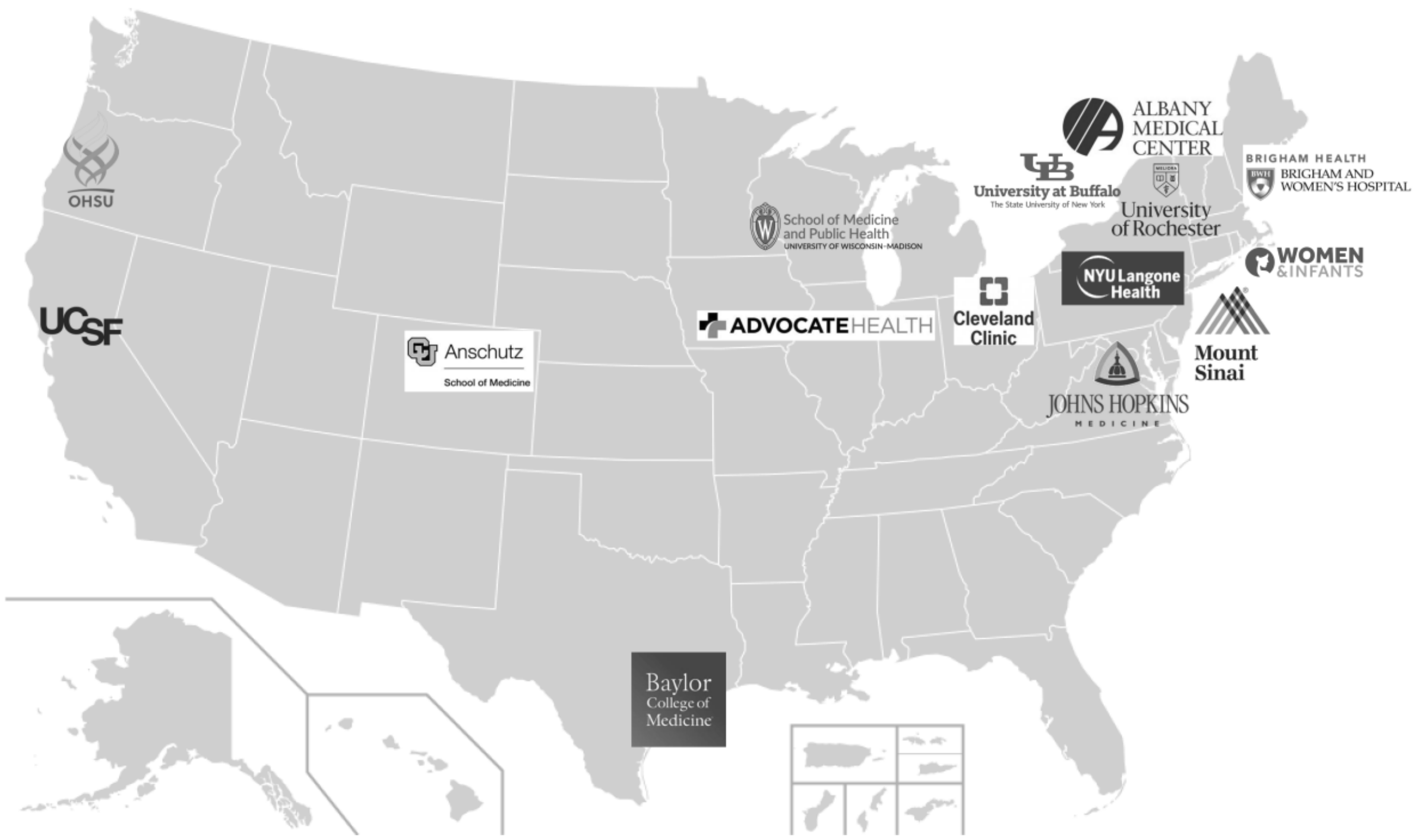

Supplement: Supplementary file 1 [file pone.0355045.s003.pdf]
