# Supplementary material for: Study protocol for first national vasa previa perinatal registry
Source: PLoS One. 2026 Aug 17;21(8):e0355045. doi: 10.1371/journal.pone.0355045 (PMC13480644; doi:10.1371/journal.pone.0355045)
Supplement: Supplementary file 2 [file pone.0355045.s001.docx]

**Supplemental Table 1**

Data Elements Collected in the Vasa Previa Without Concurrent Placenta Previa Multicenter Registry.

**Demographics**

Maternal age at delivery

Height

Weight

Race

Ethnicity

Gravidity and Parity

**Maternal Medical History**

Pregnancy conceived through assisted reproductive technology (ART). If yes, type of ART

Prior C-section? If yes, number

Prior myomectomy?

Prior Uterine septum resection?

Prior cervical surgery?

Other uterine instrumentation?

**Site information**

How many L&D units are included at your site where patients with vasa previa are admitted/delivered?

Level of maternal care per L&D unit

Level of neonatal care per L&D unit

Is there 24/7 resident coverage of unit, per unit

Is your antepartum unit located on the same floor of the hospital as your L&D unit, per L&D unit

Describe the logistics of moving patient from antepartum to L&D for urgent delivery

**Ultrasound Data**

Number of fetuses ~~gestations~~

Type of ultrasound machine used

Type of ultrasound transducer used (if known)

Type of ultrasound done (viability, dating, nuchal translucency, anatomic, follow-up anatomy, follow-up growth, other)

Was a transvaginal ultrasound done?

Gestational age at time of first ultrasound exam showing vasa previa

Type of vasa previa found on ultrasound (Type 1, Type 2, Type 3, low-lying fetal vessels, type not documented, other)

What vessel(s) was involved in the vasa previa (select all which apply). Umbilical artery~~, umbilical arteries~~, umbilical vein, unknown)

What was the diameter (mm) of each vessel involved in the vasa previa (if measured/known)

Placental location (anterior, posterior, lateral, fundal, other)

Was an accessory placental lobe present? If so, which type (succenturiate, bilobed, other)

Where was the location of the accessory placental lobe?

Type of umbilical cord insertion into the placenta (normal, marginal, velamentous, other)

Is placenta previa or low-lying placental present on the current ultrasound?

Distance from placental edge to internal cervical os

Distance between fetal vessels and internal cervical os (if measured)

Was cervical length measurement performed?

Cervical length measurement

Was pulsed Doppler assessment for the fetal vessel performed?

Additional ultrasound done?

Did vasa previa resolve on subsequent ultrasound exams?

How was resolution of the vasa previa defined? Please describe

Was a procedure performed on this vasa previa (ie. Laser coagulation)? If so, describe.

Gestational age at vasa previa resolution (weeks and days gestation)

**Antenatal management**

Was patient admitted during the antenatal period for any reason during their pregnancy?

How many hospital antepartum admissions did the patient have total?

What were the reasons for hospital antenatal admission during their pregnancy?

Gestational age at hospital admission

Where was the patient admitted? (L&D unit, antepartum unit, intensive care unit, other)

Type of antenatal testing

How often did the patient get non-stress tests during admission?

Describe NST frequency if you selected “other”

How often did the patient get a biophysical profile during admission?

Describe biophysical profile frequency if you selected “other”

Describe other antenatal testing that was done

How often did the patient get growth ultrasound during admission?

Did the patient have any of the following pregnancy-related complications?

How many bleeding episodes did the patient have during the pregnancy after the vasa previa was diagnosed?

Describe any other pertinent aspects of the patient’s antenatal hospitalization

**Delivery**

Gestational age

Mode of delivery

Was this an emergent delivery

What was the indication for delivery (preterm labor, preterm prelabor rupture of membranes, vaginal bleeding, non-reassuring fetal heart rate tracing)

How many doses/days of antenatal corticosteroids did the patient receive prior to delivery?

Was magnesium sulfate administered for fetal neuroprotection prior to delivery?

Did the patient receive tocolytics?

Did operative notes or delivery summary remark on gross examination of placenta and cord, were they consistent with vasa previa?

Placental pathology report

Did the patient have a hysterectomy at the time of delivery?

Was there a maternal death?

Was there severe maternal morbidity (as defined by CDC)

What was the overall maternal length of stay (# days) in the hospital during this admission

Did the patient have an Edinburgh Perinatal Depression Scale done at the 6–week postpartum appointment? Is yes, score

Did the patient get referred to behavioral health in the postpartum period at or before 6-week postpartum visit?

Is it documented in the patient’s postpartum notes that they suffered from traumatic birth or post-traumatic stress disorder. Acute stress reactions?

Do postpartum notes document any impact of hospitalization or delivery on the patient’s life?

**Neonatal Outcomes (per fetus)**

Was there a fetal death in utero diagnosed for this fetus?

Gestational age at fetal demise diagnosis

Pertinent details related to etiology of fetal demise in utero (if known) based on placental pathology autopsy

Neonatal birthweight in grams

Apgar score at 1 and 5 minutes

Umbilical cord blood gas drawn?

Please input the umbilical cord blood pH here

Umbilical cord blood gas origin (arterial preferred

Where was the newborn admitted after birth?

Was the newborn later transferred to the NICU?

Day of life when NICU transfer occurred

Reason for NICU transfer

Was the newborn hemoglobin level drawn within the first 48 hours of life? If so, what is the level

Did the neonate require a blood transfusion within 72 hours of birth?

Did the neonate require assisted ventilation immediately following delivery? (within 0 to 6 hours of birth)

What was the newborn’s disposition after NICU admission?

Please describe circumstances of neonatal death

What was the total number of days the newborn spent in an intensive care unit after birth?

What is the total number of days the newborn spent in the hospital, from day of delivery to the day of discharge?

Did the neonate have radiographic evidence of an intraventricular hemorrhage (IVH)? If yes, IVH grade

Did the neonate develop a diagnosis of bronchopulmonary dysplasia (BPD)? If yes, what was the severity

Was the neonate diagnosed with necrotizing enterocolitis? If yes what stage

Was the newborn discharge on respiratory support?

Was the newborn discharged on feeding support?

Please discuss any other pertinent complications occurring during the newborn period

**Recurrence**

Did the patient have another pregnancy?

Did this patient have recurrent vasa previa in subsequent pregnancy?

This table summarizes all data elements collected in the multicenter registry. Variables collected per fetus (Fetus A, B, and C in multiple gestations) use identical definitions and coding unless otherwise specified. Free-text variables are noted but not exhaustively itemized. Coding reflects the original REDCap instrument.
