# Supplementary material for: Study protocol for first national vasa previa perinatal registry
Source: PLoS One. 2026 Aug 17;21(8):e0355045. doi: 10.1371/journal.pone.0355045 (PMC13480644; doi:10.1371/journal.pone.0355045)
Supplement: Supplementary file 3 [file pone.0355045.s002.docx]

**Supplemental Table 2.** Multistep Case Ascertainment Strategy for Identification of Vasa Previa

| **Step** | **Data Source** | **Ascertainment Method** | **Purpose / Rationale** |
| --- | --- | --- | --- |
| **1** | Electronic medical record (EMR) | Identification of potential cases using diagnosis codes, problem lists, and ultrasound report keywords related to vasa previa | To capture all possible vasa previa cases with high sensitivity |
| **2** | Prenatal ultrasound reports | Review of second- and third-trimester ultrasound reports documenting suspected or confirmed vasa previa, low-lying fetal vessels, velamentous cord insertion, or abnormal placental morphology | To identify cases based on imaging findings rather than diagnosis codes alone |
| **3** | Maternal–fetal medicine (MFM) consultation notes | Manual chart review of specialist notes describing suspected or confirmed vasa previa and management plans | To corroborate imaging findings and clinical diagnosis |
| **4** | Delivery records and operative reports | Review of cesarean delivery reports for intraoperative confirmation of vasa previa or low-lying fetal vessels | To confirm diagnosis at delivery when available |
| **5** | Placental pathology reports | Review of placental examination documenting velamentous cord insertion, fetal vessels within membranes, or placental anomalies consistent with vasa previa | To provide anatomic confirmation and reduce misclassification |
| **6** | Local investigator adjudication | Independent confirmation by site investigators using predefined diagnostic criteria | To ensure consistency of case definition across institutions |
| **7** | Central study team review | Secondary review of submitted cases with resolution of discrepancies through consensus | To standardize final case inclusion across sites |
