# Supplementary material for: Study protocol for first national vasa previa perinatal registry
Source: PLoS One. 2026 Aug 17;21(8):e0355045. doi: 10.1371/journal.pone.0355045 (PMC13480644; doi:10.1371/journal.pone.0355045)
Supplement: Supplementary file 4 [file pone.0355045.s005.docx]

Dear editors of Plos One,

Thank you to the editors and reviewers for taking the time to review our Manuscript, *“Study Protocol for First National Vasa Previa Perinatal Registry”* and provide us with the opportunity to submit a revised draft. We appreciate you taking the time to carefully review our manuscript and provide insightful comments and feedback. We have revised the manuscript and provided responses below to the comments that were provided.

Reviewer #1, Point #1

A. Dr. Oyeles’s affiliation is incorrect. Please review. Also need to correct in the figure with the US map and institutions.
B. Thank you so much for drawling attention to this. We have changed his affiliation to the correct institution, Beth Isreal Deaconess Medical Center. As for the map, we removed the logos and replaced all institutions to stars to represent their location, so we didn’t have to make any adjustments as both institutions are in Boston.
C. Line 35

Reviewer #1, Point #2

A. Was there any rational behind including only centers in the US? If so what was it?
B. Thank you for this great question! There continues to be significant variability in management of vasa previa not only in the United States but around the world. We wanted to focus this initial research on the United States as these centers base care pathways on the same national guidelines from The American College of Obstetricians and Gynecologists and Society for Maternal Fetal Medicine. When we started this registry, it was largely through existing connections at various institutions across the United States. Additionally, to be approved by our Institutional Board Review, all sites had to complete a similar IRB process at their respective institutions. As the registry grows, and if we are able to obtain funding, we would like to include sites from across the world.

Reviewer #1, Point #3

A. How do you define viability?
B. This is a great point that warrants further clarification. The definition of viability for data collection is determined by each respective institution since there is variation in practice within the United States. Our institution uses 23 weeks for viability while other institutions may use 22 weeks. We plan to define viability for the whole cohort after full data collection.
C. Please see the adjusts to the language on line 436-438

Reviewer #1, Point #4

A. Ideally, this number should be agreed upon and specified in this protocol.
B. We agree that there should be an agreed upon number of case contributions to achieve access to the database for research. If sites contribute more than 20 patients, they are eligible to use data from the registry for research as long as the registry is properly cited.
C. Language was changed on line 587 to reflect this cut off.

Reviewer #1, Point #5

A. Is there a possibility that other centers that were not originally included who meet criteria for participation could join and contribute cases retrospectively or prospectively? What about centers in other countries?
B. Our hope is that this can become a living registry, which means that additional institutions that were not included in the initial registry would have the opportunity to contribute data once they receive IRB approval. Data contribution could either be retrospective or prospective based on the institution's abilities. At this time all participating institutions are located within the United States; however, in the future we may be interested in becoming an international registry. The capabilities of our registry are currently limited without a funding source.
C. Please see adjusts made to lines 597-600 to reflect the above.

Reviewer #1, Point #6

A. Is there a minimum of cases required to participate?
B. There is no minimum number of cases required to participate in the registry. However, as mentioned above, if sites would like access to the dataset they need to contribute at least 20 cases.
C. Edits were made to the caption of Table 2

Reviewer #1, Point #7

A. Consider modifying the wording to improve clarity. Do you mean number of fetuses?
B. Thank you for this clarification. Yes, we mean number of fetuses. The wording on the table has been changed.
C. Edits made to Supplemental Table 1

Reviewer #1, Point #8

A. Umbilical artery is mentioned twice. Is this intentional?
B. Yes, we meant to indicate whether one or both umbilical arteries are involved in the vasa previa.
C. Supplemental Table 1 was not changed.

Reviewer #1, Point #9

A. Might be helpful to catch how many of these patients had placenta previa that resolved and turned into a vasa previa
B. Thank you for bringing this to our attention. Our initial goal of the dataset was to exclude patients with placenta previa with concurrent vasa previa to better understand vasa previa alone. We are including patients who have resolved placenta previa or low lying vessels; therefore, we do hope to capture this data.
C. On line 343 we specified that we excluded patients that had placenta previa at the time of delivery. We then added a line 344-345 to make it clear that we included patients that had resolved placenta previa.

Reviewer #1, Point #10

A. What about asking about use of 3D rendering modalities. It would also be interesting to know how many of these cases where identified in the midsagittal position verses on lateral sweeps, and whether practitioners routinely swept the entire lower uterine segment.
B. We agree that it would be very interesting to extract additional data on how the ultrasounds were performed; however, that is outside the capability of the registry at this time. There is significant variation in how ultrasound data is recorded at each institution and it would not be possible to record this. We hope with additional future funding that prospective data collection regarding this could be incorporated into the registry.

Reviewer #1, Point #11

A. Although not recommended as a routine imaging modality, some centers consider fetal MRI to MAP vessels, particularly those who offer fetoscopic laser photocoagulation. Consider asking whether this modality of imaging was also considered during the work up.
B. To our knowledge, none of the current sites are using fetal MRI to MAP vessels. However, we did provide text boxes within RedCap to catch answers that did not fit into the answers provided. This would be where investigators can include whether nontraditional imaging modalities were used.

Reviewer 2
Biggest data set

Centers not geographically dispersed

There are several important methodological concerns that should be addressed more explicitly. These primarily relate to retrospective diagnostic ascertainment, exposure definition heterogeneity, referral-center selection bias, and temporal variability in diagnostic and management practices. These issues do not negate the value of the registry, but they substantially influence how findings should be interpreted and should be more clearly emphasized throughout the manuscript.

Abstract:
no comments

Introduction:
Reviewer #2, Point #1

A. The introduction is comprehensive and clinically informed, but it is somewhat overly expansive and reads in parts like a narrative review rather than a tightly focused justification for the registry. The epidemiologic background, screening recommendations, and survival statistics are somewhat detailed relative to their necessity for framing the central gap. This dilutes the focus on the primary methodological problem the registry is intended to solve: the absence of large, systematically collected multicenter datasets with standardized phenotyping.

Response: We appreciate this thoughtful comment and agree that the original introduction placed greater emphasis on providing a broad clinical overview than on establishing the specific rationale for the registry. We have substantially revised the introduction to improve focus and concision by reducing detailed discussion of epidemiology, screening recommendations, management controversies, and emerging therapies. The revised introduction now more directly emphasizes the principal knowledge gap motivating this study: the absence of large, multicenter datasets with standardized phenotyping capable of comprehensively characterizing vasa previa, its natural history, management, and outcomes.

Reviewer #2, Point #2
A. The most important limitation of this registry is the retrospective nature of case ascertainment across a prolonged study period (2011–2023), during which diagnostic criteria, ultrasound practices, and clinical awareness of vasa previa evolved significantly.
Cases were identified based on local clinical diagnosis and retrospective chart abstraction, rather than standardized prospective imaging criteria or centralized adjudication. As a result, there is likely substantial heterogeneity in how vasa previa was defined and recorded across institutions and over time.
This issue is particularly important given ongoing controversy in the field regarding:
-the appropriate distance threshold from the internal cervical os (e.g., ≤2 cm),
-and the classification of “low-lying fetal vessels” (2–5 cm).

Response: We agree with this important observation and agree that temporal and institutional variation in the diagnosis of vasa previa represents an important limitation of this registry. However, this registry does shed light on the extent of heterogeneity in clinical practice which is important for information gathering. We have revised the strengths and limitations to explicitly acknowledge that cases were identified based on the treating institution's clinical diagnosis and retrospective chart abstraction rather than centralized imaging review or standardized prospective diagnostic criteria. We also note that the study period spans years during which evolving ultrasound practices, increasing clinical awareness, and ongoing debate regarding diagnostic thresholds—including the distance of fetal vessels from the internal cervical os and the classification of low-lying fetal vessels—may have contributed to heterogeneity in case identification across centers. Because this registry was designed to capture real-world clinical practice across multiple institutions, we believe this variability reflects contemporary practice patterns while also highlighting the need for future prospective studies using standardized imaging definitions and centralized adjudication.

Reviewer #2, Point #3

Biggest data set

Centers not geographically dispersed

A. Although the registry includes multiple geographically distributed institutions, participating sites appear to be predominantly referral centers with not many community sites, in addition majority of the sites are in the North East which may have similar patterns. As such, the cohort should not be interpreted as nationally representative. The manuscript occasionally implies broader generalizability than the study design supports. The authors should more consistently frame this as a referral-center registry and temper any language suggesting national representativeness or population-level inference.
   It may also be useful to provide additional institutional characterization (e.g., delivery volume, referral catchment, academic status) to better contextualize the cohort.

Response:

We thank the reviewer for this thoughtful comment and agree that the registry should be interpreted as a multicenter referral-center registry rather than a nationally representative population-based cohort. We have revised the manuscript to temper language regarding generalizability and to clarify that our findings primarily reflect practice patterns at tertiary referral centers.

At the same time, because vasa previa is a rare obstetric condition that is frequently diagnosed, referred, and managed at tertiary care centers with specialized maternal-fetal medicine expertise, inclusion of academic referral centers was essential to accrue participants. Despite this limitation, the registry represents the largest multicenter dataset of pregnancies complicated by vasa previa without concurrent placenta previa assembled to date, providing an important opportunity to characterize this uncommon condition across diverse referral practices. We agree that these findings should not be interpreted as population-level estimates but rather as contemporary experience from specialized centers caring for the majority of affected patients.

Reviewer #2, Point #4

Sub analysis by year

Sensitivity analysis citing the SMFM PB and management

A. The study period spans more than a decade during which there were substantial changes in:
   -ultrasound technology and resolution,
   -screening practices for cord insertion and placental anatomy
   -universal screening
   -antenatal management strategies.
   As a result, observed variation in diagnosis and management may reflect temporal changes rather than true institutional differences.

   In addition, the manuscript itself highlights ongoing controversy regarding the appropriate sonographic definition of vasa previa, particularly with respect to vessels located between 2 and 5 cm from the internal cervical os. Because the registry relies on local clinical diagnosis and retrospective chart abstraction rather than centralized imaging review using standardized contemporary criteria, there is likely substantial temporal and interobserver heterogeneity in exposure classification.
   These entities may represent distinct clinical phenotypes with differing risk profiles. At minimum, these groups should be analyzed separately with predefined subgroup analyses. The rationale for combining or separating them should be more explicitly stated.

Response:

We agree that ultrasound technology, screening practices, diagnostic criteria, and clinical management evolved over the study period and have expanded the discussion to acknowledge these temporal changes as an important limitation of this retrospective registry.

Regarding subgroup analyses by year and by sonographic definition (classic vasa previa versus low-lying fetal vessels), these analyses are beyond the scope of the current descriptive registry manuscript. Additionally, detailed measurements of vessel distance from the internal cervical os were not consistently available across participating sites. Furthermore, annual subgroup analyses would result in relatively small sample sizes within each year, limiting statistical power and reducing the interpretability of any observed temporal differences. We agree that these represent important questions and anticipate that future analyses from the registry, using subsets of patients with complete imaging data, will specifically examine temporal trends and differences between these phenotypes.

Reviewer #2, Point #5

A. Although the registry is well designed for descriptive epidemiology, comparisons of management strategies (e.g., inpatient vs outpatient care, timing of delivery) will be highly susceptible to, confounding by indication, center-level practice effects, and unmeasured severity differences.
   Therefore, conclusions regarding optimal management strategies should be framed cautiously. The registry is best suited for hypothesis generation rather than causal inference. While this is the best data that will be available; language suggesting that the registry will directly “optimize” care or define best practices should be moderated.

Response:

We have revised the manuscript to clarify that the registry is intended primarily for descriptive analyses and hypothesis generation rather than causal inference. We also moderated language suggesting that the registry will define optimal management or best practices, instead emphasizing that it will strengthen the evidence base and inform future prospective studies.
