# Supplementary material for: Study protocol for first national vasa previa perinatal registry
Source: PLoS One. 2026 Aug 17;21(8):e0355045. doi: 10.1371/journal.pone.0355045 (PMC13480644; doi:10.1371/journal.pone.0355045)
Supplement: Peer review history

# Peer review: Decision Letter 0

**DOI:** 10.1371/journal.pone.0355045.r001

**Authors:** Alireza Abdollah Shamshirsaz

27 May 2026

Dear Dr. Toscano,

Thank you for submitting your manuscript to PLOS ONE. After careful consideration, we feel that it has merit but does not fully meet PLOS ONE’s publication criteria as it currently stands. Therefore, we invite you to submit a revised version of the manuscript that addresses the points raised during the review process.

Please submit your revised manuscript by Jul 11 2026 11:59PM. If you will need more time than this to complete your revisions, please reply to this message or contact the journal office at plosone@plos.org. When you're ready to submit your revision, log on to https://www.editorialmanager.com/pone/ and select the 'Submissions Needing Revision' folder to locate your manuscript file.

- A letter that responds to each point raised by the academic editor and reviewer(s). You should upload this letter as a separate file labeled 'Response to Reviewers'.
- A marked-up copy of your manuscript that highlights changes made to the original version. You should upload this as a separate file labeled 'Revised Manuscript with Track Changes'.
- An unmarked version of your revised paper without tracked changes. You should upload this as a separate file labeled 'Manuscript'.

If you would like to make changes to your financial disclosure, please include your updated statement in your cover letter. Guidelines for resubmitting your figure files are available below the reviewer comments at the end of this letter.

If applicable, we recommend that you deposit your laboratory protocols in protocols.io to enhance the reproducibility of your results. Protocols.io assigns your protocol its own identifier (DOI) so that it can be cited independently in the future. For instructions see: https://journals.plos.org/plosone/s/submission-guidelines#loc-laboratory-protocols. Additionally, PLOS ONE offers an option for publishing peer-reviewed Lab Protocol articles, which describe protocols hosted on protocols.io. Read more information on sharing protocols at https://plos.org/protocols?utm_medium=editorial-email&utm_source=authorletters&utm_campaign=protocols.

As the corresponding author, your ORCID iD is verified in the submission system and will appear in the published article. PLOS supports the use of ORCID, and we encourage all coauthors to register for an ORCID iD and use it as well. Please encourage your coauthors to verify their ORCID iD within the submission system before final acceptance, as unverified ORCID iDs will not appear in the published article. *Only*  the individual author can complete the verification step; PLOS staff *cannot*  verify ORCID iDs on behalf of authors.

We look forward to receiving your revised manuscript.

Kind regards,

Alireza Abdollah Shamshirsaz

Academic Editor

PLOS One

Journal requirements:

When submitting your revision, we need you to address these additional requirements.

1. Please ensure that your manuscript meets PLOS ONE's style requirements, including those for file naming. The PLOS ONE style templates can be found at

https://journals.plos.org/plosone/s/file?id=wjVg/PLOSOne_formatting_sample_main_body.pdf and

https://journals.plos.org/plosone/s/file?id=ba62/PLOSOne_formatting_sample_title_authors_affiliations.pdf

2. Please provide additional details regarding participant consent. In the ethics statement in the Methods and online submission information, please ensure that you have specified (1) whether consent was informed and (2) what type you obtained (for instance, written or verbal, and if verbal, how it was documented and witnessed). If your study included minors, state whether you obtained consent from parents or guardians. If the need for consent was waived by the ethics committee, please include this information.

If you are reporting a retrospective study of medical records or archived samples, please ensure that you have discussed whether all data were fully anonymized before you accessed them and/or whether the IRB or ethics committee waived the requirement for informed consent. If patients provided informed written consent to have data from their medical records used in research, please include this information.

3. Please include a separate caption for each figure in your manuscript.

4. We note that Figure 1 in your submission contain [map/satellite] images which may be copyrighted. All PLOS content is published under the Creative Commons Attribution License (CC BY 4.0), which means that the manuscript, images, and Supporting Information files will be freely available online, and any third party is permitted to access, download, copy, distribute, and use these materials in any way, even commercially, with proper attribution. For these reasons, we cannot publish previously copyrighted maps or satellite images created using proprietary data, such as Google software (Google Maps, Street View, and Earth). For more information, see our copyright guidelines: http://journals.plos.org/plosone/s/licenses-and-copyright.

We require you to either (1) present written permission from the copyright holder to publish these figures specifically under the CC BY 4.0 license, or (2) remove the figures from your submission:

1. You may seek permission from the original copyright holder of Figure 1 to publish the content specifically under the CC BY 4.0 license.

We recommend that you contact the original copyright holder with the Content Permission Form (http://journals.plos.org/plosone/s/file?id=7c09/content-permission-form.pdf) and the following text:

“I request permission for the open-access journal PLOS ONE to publish XXX under the Creative Commons Attribution License (CCAL) CC BY 4.0 (http://creativecommons.org/licenses/by/4.0/). Please be aware that this license allows unrestricted use and distribution, even commercially, by third parties. Please reply and provide explicit written permission to publish XXX under a CC BY license and complete the attached form.”

Please upload the completed Content Permission Form or other proof of granted permissions as an "Other" file with your submission.

In the figure caption of the copyrighted figure, please include the following text: “Reprinted from [ref] under a CC BY license, with permission from [name of publisher], original copyright [original copyright year].”

2. If you are unable to obtain permission from the original copyright holder to publish these figures under the CC BY 4.0 license or if the copyright holder’s requirements are incompatible with the CC BY 4.0 license, please either i) remove the figure or ii) supply a replacement figure that complies with the CC BY 4.0 license. Please check copyright information on all replacement figures and update the figure caption with source information. If applicable, please specify in the figure caption text when a figure is similar but not identical to the original image and is therefore for illustrative purposes only.

The following resources for replacing copyrighted map figures may be helpful:

USGS National Map Viewer (public domain): http://viewer.nationalmap.gov/viewer/

The Gateway to Astronaut Photography of Earth (public domain): http://eol.jsc.nasa.gov/sseop/clickmap/

Maps at the CIA (public domain): https://www.cia.gov/library/publications/the-world-factbook/index.html and https://www.cia.gov/library/publications/cia-maps-publications/index.html

NASA Earth Observatory (public domain): http://earthobservatory.nasa.gov/

Landsat: http://landsat.visibleearth.nasa.gov/

USGS EROS (Earth Resources Observatory and Science (EROS) Center) (public domain): http://eros.usgs.gov/#

Natural Earth (public domain): http://www.naturalearthdata.com/

5. We notice that your supplementary tables are included in the manuscript file. Please remove them and upload them with the file type 'Supporting Information'. Please ensure that each Supporting Information file has a legend listed in the manuscript after the references list.

If the reviewer comments include a recommendation to cite specific previously published works, please review and evaluate these publications to determine whether they are relevant and should be cited. There is no requirement to cite these works unless the editor has indicated otherwise.

Please review your reference list to ensure that it is complete and correct. If you have cited papers that have been retracted, please include the rationale for doing so in the manuscript text, or remove these references and replace them with relevant current references. Any changes to the reference list should be mentioned in the rebuttal letter that accompanies your revised manuscript. If you need to cite a retracted article, indicate the article’s retracted status in the References list and also include a citation and full reference for the retraction notice.

[Note: HTML markup is below. Please do not edit.]

Reviewers' comments:

Reviewer's Responses to Questions

**Comments to the Author**

1. Does the manuscript provide a valid rationale for the proposed study, with clearly identified and justified research questions?

Reviewer #1: Yes

Reviewer #2: Yes

**********

2. Is the protocol technically sound and planned in a manner that will lead to a meaningful outcome and allow testing the stated hypotheses?

Reviewer #1: Yes

Reviewer #2: Yes

**********

3. Is the methodology feasible and described in sufficient detail to allow the work to be replicable?

Reviewer #1: Yes

Reviewer #2: Yes

**********

4. Have the authors described where all data underlying the findings will be made available when the study is complete?

The PLOS Data policy

Reviewer #1: Yes

Reviewer #2: Yes

**********

5. Is the manuscript presented in an intelligible fashion and written in standard English?

Reviewer #1: Yes

Reviewer #2: Yes

**********

Please use the space provided to explain your answers to the questions above and, if applicable, provide comments about issues authors must address before this protocol can be accepted for publication. You may also include additional comments for the author, including concerns about research or publication ethics.

You may also provide optional suggestions and comments to authors that they might find helpful in planning their study.

Reviewer #1: Congratulations on this long-awaited initiative. I find this project highly relevant and am looking forward to seeing the results. I have included my suggestions as comments in the PDF file provided for review.

Reviewer #2: This manuscript describes the development and implementation of a multicenter U.S. registry of pregnancies complicated by vasa previa without concurrent placenta previa. The study addresses an important gap in obstetric literature, as vasa previa is a rare but high-risk condition for which large, systematically collected datasets are limited. The effort to coordinate 17 institutions and construct a standardized clinical registry is commendable and represents a meaningful contribution to the field.

The manuscript is generally well written, methodologically detailed, and clearly structured. The registry infrastructure, data governance framework, and case ascertainment approach are strengths of the submission. The dataset has the potential to generate valuable descriptive insights into variation in diagnosis, management, and outcomes.

However, there are several important methodological concerns that should be addressed more explicitly. These primarily relate to retrospective diagnostic ascertainment, exposure definition heterogeneity, referral-center selection bias, and temporal variability in diagnostic and management practices. These issues do not negate the value of the registry, but they substantially influence how findings should be interpreted and should be more clearly emphasized throughout the manuscript.

Abstract:

no comments

Introduction:

The introduction is comprehensive and clinically informed, but it is somewhat overly expansive and reads in parts like a narrative review rather than a tightly focused justification for the registry. The epidemiologic background, screening recommendations, and survival statistics are somewhat detailed relative to their necessity for framing the central gap. This dilutes the focus on the primary methodological problem the registry is intended to solve: the absence of large, systematically collected multicenter datasets with standardized phenotyping.

-The most important limitation of this registry is the retrospective nature of case ascertainment across a prolonged study period (2011–2023), during which diagnostic criteria, ultrasound practices, and clinical awareness of vasa previa evolved significantly.

Cases were identified based on local clinical diagnosis and retrospective chart abstraction, rather than standardized prospective imaging criteria or centralized adjudication. As a result, there is likely substantial heterogeneity in how vasa previa was defined and recorded across institutions and over time.

This issue is particularly important given ongoing controversy in the field regarding:

-the appropriate distance threshold from the internal cervical os (e.g., ≤2 cm),

-and the classification of “low-lying fetal vessels” (2–5 cm).

Although the registry includes multiple geographically distributed institutions, participating sites appear to be predominantly referral centers with not many community sites, in addition majority of the sites are in the North East which may have similar patterns. As such, the cohort should not be interpreted as nationally representative. The manuscript occasionally implies broader generalizability than the study design supports. The authors should more consistently frame this as a referral-center registry and temper any language suggesting national representativeness or population-level inference.

It may also be useful to provide additional institutional characterization (e.g., delivery volume, referral catchment, academic status) to better contextualize the cohort.

The study period spans more than a decade during which there were substantial changes in:

-ultrasound technology and resolution,

-screening practices for cord insertion and placental anatomy

-universal screening

-antenatal management strategies.

As a result, observed variation in diagnosis and management may reflect temporal changes rather than true institutional differences.

In addition, the manuscript itself highlights ongoing controversy regarding the appropriate sonographic definition of vasa previa, particularly with respect to vessels located between 2 and 5 cm from the internal cervical os. Because the registry relies on local clinical diagnosis and retrospective chart abstraction rather than centralized imaging review using standardized contemporary criteria, there is likely substantial temporal and interobserver heterogeneity in exposure classification.

These entities may represent distinct clinical phenotypes with differing risk profiles. At minimum, these groups should be analyzed separately with predefined subgroup analyses. The rationale for combining or separating them should be more explicitly stated.

Although the registry is well designed for descriptive epidemiology, comparisons of management strategies (e.g., inpatient vs outpatient care, timing of delivery) will be highly susceptible to, confounding by indication, center-level practice effects, and unmeasured severity differences.

Therefore, conclusions regarding optimal management strategies should be framed cautiously. The registry is best suited for hypothesis generation rather than causal inference. While this is the best data that will be available; language suggesting that the registry will directly “optimize” care or define best practices should be moderated.

**********

what does this mean?). If published, this will include your full peer review and any attached files.

If you choose “no”, your identity will remain anonymous but your review may still be made public.

**Do you want your identity to be public for this peer review?**  For information about this choice, including consent withdrawal, please see our Privacy Policy

Reviewer #1: No

Reviewer #2: No

**********

[NOTE: If reviewer comments were submitted as an attachment file, they will be attached to this email and accessible via the submission site. Please log into your account, locate the manuscript record, and check for the action link "View Attachments". If this link does not appear, there are no attachment files.]

To ensure your figures meet our technical requirements, please review our figure guidelines: https://journals.plos.org/plosone/s/figures

You may also use PLOS’s free figure tool, NAAS, to help you prepare publication quality figures: https://journals.plos.org/plosone/s/figures#loc-tools-for-figure-preparation.

NAAS will assess whether your figures meet our technical requirements by comparing each figure against our figure specifications.

Attachment Submitted filename: Reviewer comments.pdf

# Author comment: Author response to Decision Letter 1

**DOI:** 10.1371/journal.pone.0355045.r002

13 Jul 2026

Dear editors of Plos One,

Thank you to the editors and reviewers for taking the time to review our Manuscript, “Study Protocol for First National Vasa Previa Perinatal Registry” and provide us with the opportunity to submit a revised draft. We appreciate you taking the time to carefully review our manuscript and provide insightful comments and feedback. We have revised the manuscript and provided responses below to the comments that were provided.

Reviewer #1, Point #1

A. Dr. Oyeles’s affiliation is incorrect. Please review. Also need to correct in the figure with the US map and institutions.

B. Thank you so much for drawling attention to this. We have changed his affiliation to the correct institution, Beth Isreal Deaconess Medical Center. As for the map, we removed the logos and replaced all institutions to stars to represent their location, so we didn’t have to make any adjustments as both institutions are in Boston.

C. Line 35

Reviewer #1, Point #2

A. Was there any rational behind including only centers in the US? If so what was it?

B. Thank you for this great question! There continues to be significant variability in management of vasa previa not only in the United States but around the world. We wanted to focus this initial research on the United States as these centers base care pathways on the same national guidelines from The American College of Obstetricians and Gynecologists and Society for Maternal Fetal Medicine. When we started this registry, it was largely through existing connections at various institutions across the United States. Additionally, to be approved by our Institutional Board Review, all sites had to complete a similar IRB process at their respective institutions. As the registry grows, and if we are able to obtain funding, we would like to include sites from across the world.

Reviewer #1, Point #3

A. How do you define viability?

B. This is a great point that warrants further clarification. The definition of viability for data collection is determined by each respective institution since there is variation in practice within the United States. Our institution uses 23 weeks for viability while other institutions may use 22 weeks. We plan to define viability for the whole cohort after full data collection.

C. Please see the adjusts to the language on line 436-438

Reviewer #1, Point #4

A. Ideally, this number should be agreed upon and specified in this protocol.

B. We agree that there should be an agreed upon number of case contributions to achieve access to the database for research. If sites contribute more than 20 patients, they are eligible to use data from the registry for research as long as the registry is properly cited.

C. Language was changed on line 587 to reflect this cut off.

Reviewer #1, Point #5

A. Is there a possibility that other centers that were not originally included who meet criteria for participation could join and contribute cases retrospectively or prospectively? What about centers in other countries?

B. Our hope is that this can become a living registry, which means that additional institutions that were not included in the initial registry would have the opportunity to contribute data once they receive IRB approval. Data contribution could either be retrospective or prospective based on the institution's abilities. At this time all participating institutions are located within the United States; however, in the future we may be interested in becoming an international registry. The capabilities of our registry are currently limited without a funding source.

C. Please see adjusts made to lines 597-600 to reflect the above.

Reviewer #1, Point #6

A. Is there a minimum of cases required to participate?

B. There is no minimum number of cases required to participate in the registry. However, as mentioned above, if sites would like access to the dataset they need to contribute at least 20 cases.

C. Edits were made to the caption of Table 2

Reviewer #1, Point #7

A. Consider modifying the wording to improve clarity. Do you mean number of fetuses?

B. Thank you for this clarification. Yes, we mean number of fetuses. The wording on the table has been changed.

C. Edits made to Supplemental Table 1

Reviewer #1, Point #8

A. Umbilical artery is mentioned twice. Is this intentional?

B. Yes, we meant to indicate whether one or both umbilical arteries are involved in the vasa previa.

C. Supplemental Table 1 was not changed.

Reviewer #1, Point #9

A. Might be helpful to catch how many of these patients had placenta previa that resolved and turned into a vasa previa

B. Thank you for bringing this to our attention. Our initial goal of the dataset was to exclude patients with placenta previa with concurrent vasa previa to better understand vasa previa alone. We are including patients who have resolved placenta previa or low lying vessels; therefore, we do hope to capture this data.

C. On line 343 we specified that we excluded patients that had placenta previa at the time of delivery. We then added a line 344-345 to make it clear that we included patients that had resolved placenta previa.

Reviewer #1, Point #10

A. What about asking about use of 3D rendering modalities. It would also be interesting to know how many of these cases where identified in the midsagittal position verses on lateral sweeps, and whether practitioners routinely swept the entire lower uterine segment.

B. We agree that it would be very interesting to extract additional data on how the ultrasounds were performed; however, that is outside the capability of the registry at this time. There is significant variation in how ultrasound data is recorded at each institution and it would not be possible to record this. We hope with additional future funding that prospective data collection regarding this could be incorporated into the registry.

Reviewer #1, Point #11

A. Although not recommended as a routine imaging modality, some centers consider fetal MRI to MAP vessels, particularly those who offer fetoscopic laser photocoagulation. Consider asking whether this modality of imaging was also considered during the work up.

B. To our knowledge, none of the current sites are using fetal MRI to MAP vessels. However, we did provide text boxes within RedCap to catch answers that did not fit into the answers provided. This would be where investigators can include whether nontraditional imaging modalities were used.

Reviewer 2

Reviewer #2, Point #1:

We appreciate this thoughtful comment and agree that the original introduction placed greater emphasis on providing a broad clinical overview than on establishing the specific rationale for the registry. We have substantially revised the introduction to improve focus and concision by reducing detailed discussion of epidemiology, screening recommendations, management controversies, and emerging therapies. The revised introduction now more directly emphasizes the principal knowledge gap motivating this study: the absence of large, multicenter datasets with standardized phenotyping capable of comprehensively characterizing vasa previa, its natural history, management, and outcomes.

Reviewer #2, Point #2:

We agree with this important observation and agree that temporal and institutional variation in the diagnosis of vasa previa represents an important limitation of this registry. However, this registry does shed light on the extent of heterogeneity in clinical practice which is important for information gathering. We have revised the strengths and limitations to explicitly acknowledge that cases were identified based on the treating institution's clinical diagnosis and retrospective chart abstraction rather than centralized imaging review or standardized prospective diagnostic criteria. We also note that the study period spans years during which evolving ultrasound practices, increasing clinical awareness, and ongoing debate regarding diagnostic thresholds—including the distance of fetal vessels from the internal cervical os and the classification of low-lying fetal vessels—may have contributed to heterogeneity in case identification across centers. Because this registry was designed to capture real-world clinical practice across multiple institutions, we believe this variability reflects contemporary practice patterns while also highlighting the need for future prospective studies using standardized imaging definitions and centralized adjudication.

Reviewer #2, Point #3:

We thank the reviewer for this thoughtful comment and agree that the registry should be interpreted as a multicenter referral-center registry rather than a nationally representative population-based cohort. We have revised the manuscript to temper language regarding generalizability and to clarify that our findings primarily reflect practice patterns at tertiary referral centers.

At the same time, because vasa previa is a rare obstetric condition that is frequently diagnosed, referred, and managed at tertiary care centers with specialized maternal-fetal medicine expertise, inclusion of academic referral centers was essential to accrue participants. Despite this limitation, the registry represents the largest multicenter dataset of pregnancies complicated by vasa previa without concurrent placenta previa assembled to date, providing an important opportunity to characterize this uncommon condition across diverse referral practices. We agree that these findings should not be interpreted as population-level estimates but rather as contemporary experience from specialized centers caring for the majority of affected patients.

Reviewer #2, Point #4

We agree that ultrasound technology, screening practices, diagnostic criteria, and clinical management evolved over the study period and have expanded the discussion to acknowledge these temporal changes as an important limitation of this retrospective registry.

Regarding subgroup analyses by year and by sonographic definition (classic vasa previa versus low-lying fetal vessels), these analyses are beyond the scope of the current descriptive registry manuscript. Additionally, detailed measurements of vessel distance from the internal cervical os were not consistently available across participating sites. Furthermore, annual subgroup analyses would result in relatively small sample sizes within each year, limiting statistical power and reducing the interpretability of any observed temporal differences. We agree that these represent important questions and anticipate that future analyses from the registry, using subsets of patients with complete imaging data, will specifically examine temporal trends and differences between these phenotypes.

Reviewer #2, Point #5

We have revised the manuscript to clarify that the registry is intended primarily for descriptive analyses and hypothesis generation rather than causal inference. We also moderated language suggesting that the registry will define optimal management or best practices, instead emphasizing that it will strengthen the evidence base and inform future prospective studies.

Attachment Submitted filename: Response to the Editors- Vasa Previa 8JULY2026.docx

# Editor report: Decision Letter 1

**DOI:** 10.1371/journal.pone.0355045.r003

**Authors:** Alireza Abdollah Shamshirsaz

16 Jul 2026

Study Protocol for First National Vasa Previa Perinatal Registry

PONE-D-26-11238R1

Dear Dr. Marika Toscano,

We’re pleased to inform you that your manuscript has been judged scientifically suitable for publication and will be formally accepted for publication once it meets all outstanding technical requirements.

Within one week, you’ll receive an e-mail detailing the required amendments. When these have been addressed, you’ll receive a formal acceptance letter and your manuscript will be scheduled for publication.

An invoice will be generated when your article is formally accepted. Please note, if your institution has a publishing partnership with PLOS and your article meets the relevant criteria, all or part of your publication costs will be covered. Please make sure your user information is up-to-date by logging into Editorial Manager at Editorial Manager® and clicking the ‘Update My Information' link at the top of the page. For questions related to billing, please contact billing support.

If your institution or institutions have a press office, please notify them about your upcoming paper to help maximize its impact. If they’ll be preparing press materials, please inform our press team as soon as possible -- no later than 48 hours after receiving the formal acceptance. Your manuscript will remain under strict press embargo until 2 pm Eastern Time on the date of publication. For more information, please contact onepress@plos.org.

Kind regards,

Alireza Abdollah Shamshirsaz

Academic Editor

PLOS One

Additional Editor Comments (optional):

Reviewers' comments:

# Editor report: Acceptance letter

**DOI:** 10.1371/journal.pone.0355045.r004

**Authors:** Alireza Abdollah Shamshirsaz

PONE-D-26-11238R1

PLOS One

Dear Dr. Toscano,

I'm pleased to inform you that your manuscript has been deemed suitable for publication in PLOS One. Congratulations! Your manuscript is now being handed over to our production team.

At this stage, our production department will prepare your paper for publication. This includes ensuring the following:

* All references, tables, and figures are properly cited

* All relevant supporting information is included in the manuscript submission,

* There are no issues that prevent the paper from being properly typeset

You will receive further instructions from the production team, including instructions on how to review your proof when it is ready. Please keep in mind that we are working through a large volume of accepted articles, so please give us a few days to review your paper and let you know the next and final steps.

Lastly, if your institution or institutions have a press office, please let them know about your upcoming paper now to help maximize its impact. If they'll be preparing press materials, please inform our press team within the next 48 hours. Your manuscript will remain under strict press embargo until 2 pm Eastern Time on the date of publication. For more information, please contact onepress@plos.org.

You will receive an invoice from PLOS for your publication fee after your manuscript has reached the completed accept phase. If you receive an email requesting payment before acceptance or for any other service, this may be a phishing scheme. Learn how to identify phishing emails and protect your accounts at https://explore.plos.org/phishing.

If we can help with anything else, please email us at customercare@plos.org.

Thank you for submitting your work to PLOS ONE and supporting open access.

Kind regards,

PLOS ONE Editorial Office Staff

on behalf of

Dr. Alireza Abdollah Shamshirsaz

Academic Editor

PLOS One
